# Executive functions in patients with bilateral and unilateral peripheral vestibular dysfunction

**DOI:** 10.1007/s00415-024-12267-7

**Published:** 2024-03-11

**Authors:** Corina G. Schöne, Dominique Vibert, Fred W. Mast

**Affiliations:** 1 Department of Psychology, University of Bern, Bern, Switzerland; 2 Department of Otorhinolaryngology, Head and Neck Surgery, Inselspital, University Hospital Bern, University of Bern, Bern, Switzerland

**Keywords:** Peripheral vestibular dysfunction, Executive function, Verbal initiation, Working memory

## Abstract

Previous research suggests that patients with peripheral vestibular dysfunction (PVD) suffer from nonspatial cognitive problems, including executive impairments. However, previous studies that assessed executive functions are conflicting, limited to single executive components, and assessments are confounded by other cognitive functions. We compared performance in a comprehensive executive test battery in a large sample of 83 patients with several conditions of PVD (34 bilateral, 29 chronic unilateral, 20 acute unilateral) to healthy controls who were pairwise matched to patients regarding age, sex, and education. We assessed basic and complex executive functions with validated neuropsychological tests. Patients with bilateral PVD performed worse than controls in verbal initiation and working memory span, while other executive functions were preserved. Patients with chronic unilateral PVD had equal executive performance as controls. Patients with acute unilateral PVD performed worse than controls in the exact same tests as patients with bilateral PVD (verbal initiation, working memory span); however, this effect in patients with acute PVD diminished after correcting for multiple comparisons. Hearing loss and affective disorders did not influence our results. Vestibular related variables (disease duration, symptoms, dizziness handicap, deafferentation degree, and compensation) did not predict verbal initiation or working memory span in patients with bilateral PVD. The results suggest that bilateral PVD not only manifests in difficulties when solving spatial tasks but leads to more general neurocognitive deficits. This understanding is important for multidisciplinary workgroups (e.g., neurotologists, neurologists, audiologists) that are involved in diagnosing and treating patients with PVD. We recommend screening patients with PVD for executive impairments and if indicated providing them with cognitive training or psychoeducational support.

**Supplementary Information:**

The online version contains supplementary material available at 10.1007/s00415-024-12267-7.

## Introduction

Patients with peripheral vestibular dysfunction (PVD) suffer from cognitive problems [11]. Cognitive problems are present even in mild PVD [104] and can persist long after a patient has clinically recovered [10, 88]. Several studies showed that patients with PVD have problems in spatial cognitive domains, such as spatial memory, spatial navigation, or mental rotation [8, 10, 20, 22, 39, 62, 65, 87]. However, recent research fosters the claim that patients with PVD also suffer from cognitive problems in nonspatial domains such as nonspatial memory, processing speed, or executive functions [19, 74].

Executive functions are a collection of cognitive processes responsible for purposeful, goal-directed behavior [37]. They include basic and complex cognitive processes. Basic executive functions include initiation, inhibition, cognitive flexibility, or working memory. Complex executive functions are for example problem solving, planning, or monitoring [3, 21, 29, 69]. Executive functions are essential for mental and physical health, academic and life success, and daily life functioning [27]. Impaired executive functions have intense negative consequences and cause a poor quality of life [99].

There are various hypotheses for cognitive problems in patients with PVD. Visuo-spatial problems have been associated with altered brain structures, especially in the hippocampus [15]. However, the nonspatial cognitive problems are unlikely due to changes in the hippocampus [88]. Alternative explanations are plasticity processes in the neocortex or the vestibular nuclei [54, 88], changed neuronal connections from the vestibular nerve to cortical areas involved in cognitive processing [32, 75], affective disorders [11], or prioritized attentional resources on maintaining balance [83].

Subjective reports indicate that patients with PVD have serious executive problems. Most patients in a group of patients with bilateral PVD complained about difficulties with daily life activities such as an inability to prioritize tasks [12] or problems with dual tasking [64]. Unexpectedly, cognitive-based daily life activities such as managing finances were more impaired than mobility-based activities [44]. Other studies focused specifically on the executive deficits that underlie worse cognitive performance in patients with PVD. For example, patients with acute neuritis performed worse in generating sequences of random numbers [70] or a math achievement task [71] than healthy controls. These problems indicate a deficit in working memory.

Behavioral measures in patients with PVD show executive deficits, but evidence is conflicting. Patients with bilateral as well as patients with chronic unilateral PVD performed worse in a general initiation task than healthy controls [75]. However, patients with bilateral PVD performed equally as healthy controls in a verbal fluency task, a subdomain of initiation [101]. Inhibition and working memory were found to be reduced in patients with bilateral, chronic unilateral, and acute unilateral PVD [1, 4, 23, 26, 31, 56, 70, 71, 75, 77, 98]. Some other studies, however, did not find reduced inhibition or working memory performance in patients with bilateral or chronic unilateral PVD [1, 23, 75, 98]. Cognitive flexibility was reduced in patients with chronic unilateral [26, 31], but normal in patients with bilateral [101] or a mixed sample of bilateral and chronic unilateral PVD [74, 98].

Previous studies that behaviorally measured executive functions in patients with PVD are limited for the following reasons: First, all mentioned studies investigated some isolated executive components instead of administering a comprehensive executive test battery. Therefore, previous studies do not allow for a general conclusion about impaired executive functions. Second, in some studies, executive components were measured with spatial tests [26, 31, 56], thus making it impossible to disentangle spatial from executive deficits. Third, some studies did not control for processing speed when assessing reaction time in executive tasks [31, 77]. Fourth, some studies refer to executive dysfunction when non-executive functions were assessed, e.g., short-term memory instead of working memory or motor speed instead of cognitive flexibility [42, 101].

We investigated whether executive performance in a large sample of patients with PVD (*n* = 83) differs from carefully pairwise matched healthy controls. We integrated several conditions of PVD (bilateral, chronic unilateral, acute unilateral). Solving limitations of previous studies, we used a comprehensive executive test battery with validated neuropsychological tests including basic and complex executive functions. Executive tests were nonspatial if possible, and we controlled for processing speed in reaction time measurements. This methodology allows thorough conclusions about executive impairments ruling out influences of impaired spatial cognition or processing speed. We also assessed subjective executive performance, the impact of PVD on daily life functioning, and as control variables  intelligence and global cognitive level. Moreover, we investigated whether non-vestibular related variables in patients and controls (hearing loss, affective disorders) and vestibular related variables in patients (disease duration, symptoms, dizziness handicap, deafferentation degree, and compensation) influenced our results.

We hypothesized that executive performance differs between patients with PVD and healthy controls. In addition, we hypothesized that non-vestibular related variables may explain the differences in executive performance between patients with PVD and healthy controls. Further, we hypothesized that vestibular related variables in patients predict executive performance.

## Methods

### Ethical considerations

The study was conducted in agreement with the Declaration of Helsinki. The study protocol was approved by the ethics committee of the Canton Bern, Switzerland. All participants gave their written informed consent prior to study participation. Participants received a compensation of 80 Swiss francs.

### Participants

#### Patients

Ninety-eight patients were recruited from the Department of Otorhinolaryngology, Head and Neck Surgery, University Hospital of Bern, Switzerland. According to the criteria of the Bárány Society [92, 94], patients suffered from either chronic bilateral (*n* = 40), chronic unilateral (*n* = 36), or acute unilateral (*n* = 22) PVD. PVD was defined as chronic if the diagnosis lasted for at least 6 months, or acute if a patient was diagnosed maximum 1 month ago [14, 93, 94]. Patients with chronic PVD (chronic bilateral and chronic unilateral) were recruited from the neurotological database, whereas patients with acute PVD were recruited at the ENT emergency station between 2018 and 2020. PVD had different etiologies: the most common etiology was idiopathic in patients with bilateral PVD (representative for bilateral PVD, see [57]), vestibular schwannoma in patients with chronic unilateral PVD, and vestibular neuritis in patients with acute PVD. Detailed diagnosis of patients included in the study is presented in “Results”. Patients were asked to participate in our study by written letter.

All patients underwent a neurotological examination with Videonystagmography (VNG, NysStar II®, Difra Instrumentation, Belgium). Vestibulo-ocular reflex was measured using the bithermal caloric test (low frequencies). The function of the six semicircular canals at high frequencies was assessed using the video head impulse test (v-HIT, ICS Impulse®, Otometrics, Denmark). The function of the otolithic organs was recorded using the click-evoked cervical, and ocular vestibular evoked myogenic potentials (c-VEMP, o-VEMP, Eclipse VEMP®, Intercoustics GmbH, Germany). The vestibulo-spinal reflex was measured with dynamic posturography (Unterberger testing, SwayStar, [2]).

Patients were excluded from the study if they fulfilled one of the following criteria: age below 18 or above 80 years; central vestibular disorders [51], peripheral polyneuropathy; serious cardiovascular, metabolic, neurologic, or degenerative disease; neuroleptics; cerebral concussion during the year prior to the study, or insufficient German language skills.

To investigate whether *vestibular related variables* influenced our main results, we measured and defined five vestibular related variables in patients: disease duration, symptoms, dizziness handicap, deafferentation degree, and compensation.

*Disease duration* was defined as the time between the diagnosis of PVD and study participation. The timepoint of diagnosis was taken from the patient medical reports.

*Symptoms* were measured by asking patients about the intensity of actual vestibular symptoms on the day of study participation. Symptoms included vertigo, vertigo in head and body movements, unsteady gait in light and darkness, fall tendency, oscillopsia, and movement sensations. Each symptom was evaluated on a Likert scale from 1 to 10.

*Dizziness handicap* quantifies the self-perceived impact of dizziness on daily life. Dizziness handicap was assessed with a German version of the Dizziness Handicap Inventory (DHI-G, [34, 53]).

*Deafferentation degree* was classified into two groups: complete or incomplete vestibular deafferentation. Complete vestibular deafferentation was defined by pathological semicircular canal and pathological otolithic functions. Incomplete vestibular deafferentation was used when semicircular canal function at low frequencies was pathological while otolithic functions remained intact. In case of unilateral PVD, areflexia of the lateral semicircular canal in caloric testing was defined as an absence of nystagmic response, and hyporeflexia as a unilateral vestibular paresis of > 20% side difference, calculated according to Jongkee’s formula. In case of bilateral PVD, areflexia was defined as an absence of nystagmic response on both sides, and a hyporeflexia as a reduced caloric response calculated according to the criteria of the Bárány Society [92]. Within v-HIT, gains of VOR < 0.8 (horizontal canals) and < 0.6 (vertical canals) were considered as decreased. Within cervical and ocular VEMPS, the presence or absence of P1 and N1 determined the presence or absence of responses, respectively.

*Vestibular compensation* was evaluated by the degree of body sway using the roll angle parameter of the dynamic posturography [2] during the task walking in place with eyes closed (Unterberger testing). The central compensation mechanism in the brain following PVD is a complex mechanism involving vestibular, visual, and somatosensory cues. The Unterberger testing corresponds to one of the clinical signs of the central compensation mechanism.

#### Healthy controls

Fifty-four healthy controls were recruited by newspaper advertisements and word of mouth. Controls were recruited pairwise comparable to each patient in sex, age, and education. Differences between a patient and a control were maximal 4 years of age (three exceptions of 5 years) and two levels in maximal reached education (four exceptions of more than two levels) (assessment of education level is described in “Procedure”). In some cases, the same healthy control served as multiple control for patients from different patient groups (e.g., the same healthy control for a bilateral and a chronic unilateral patient). This procedure allowed us to have as few controls as necessary. Pairwise matches of patients and controls are presented in the Supplementary Material (Supplementary Table S1). We created three different control groups for the three patient groups (bilateral controls, chronic unilateral controls, acute unilateral controls). Using this careful pairwise matching strategy, we can minimize demographic influences on executive performance. Controls had no past neurotological disease. To exclude present vestibular dysfunction, controls underwent a neurotological examination. The neurotological examination included the same tests as for patients (described above). Three controls preferred not to undergo the neurotological examination. They answered screening questions of PVD instead and indicated no vestibular related problems. Exclusion criteria for healthy participants were the same as in patients (described above).

### Procedure

The neuropsychological examination lasted 90–120 min with a break halfway of the test battery. The order of the neuropsychological tests was the same for each participant. Tests requiring a high need for concentration were placed at the beginning and after the break. Examiners had at least bachelor’s degree in Psychology and were intensively trained in doing the neuropsychological assessments in a highly standardized manner. Standardized assessments are crucial to exclude subjective influences of examiners on the evaluation of neuropsychological performance.

Demographic variables and non-vestibular related variables were quantified with paper pencil questions. *Education* was measured as the maximal education reached (0 = primary education; 1 = secondary education, first level; 2 = secondary education, second level; 3 = apprenticeship or further training; 4 = high school; 5 = bachelor’s degree; 6 = master’s degree; 7 = Ph.D.). The classification of education in gradual levels rather than years of schooling is considered to better assess the link between education and the capacity of the brain to cope with neuropathology or age-related changes [35]. To assess *non-vestibular related variables*, we asked participants in written form about hearing loss and psychological disorders.

### Neuropsychological assessment

**Fig. 1:**
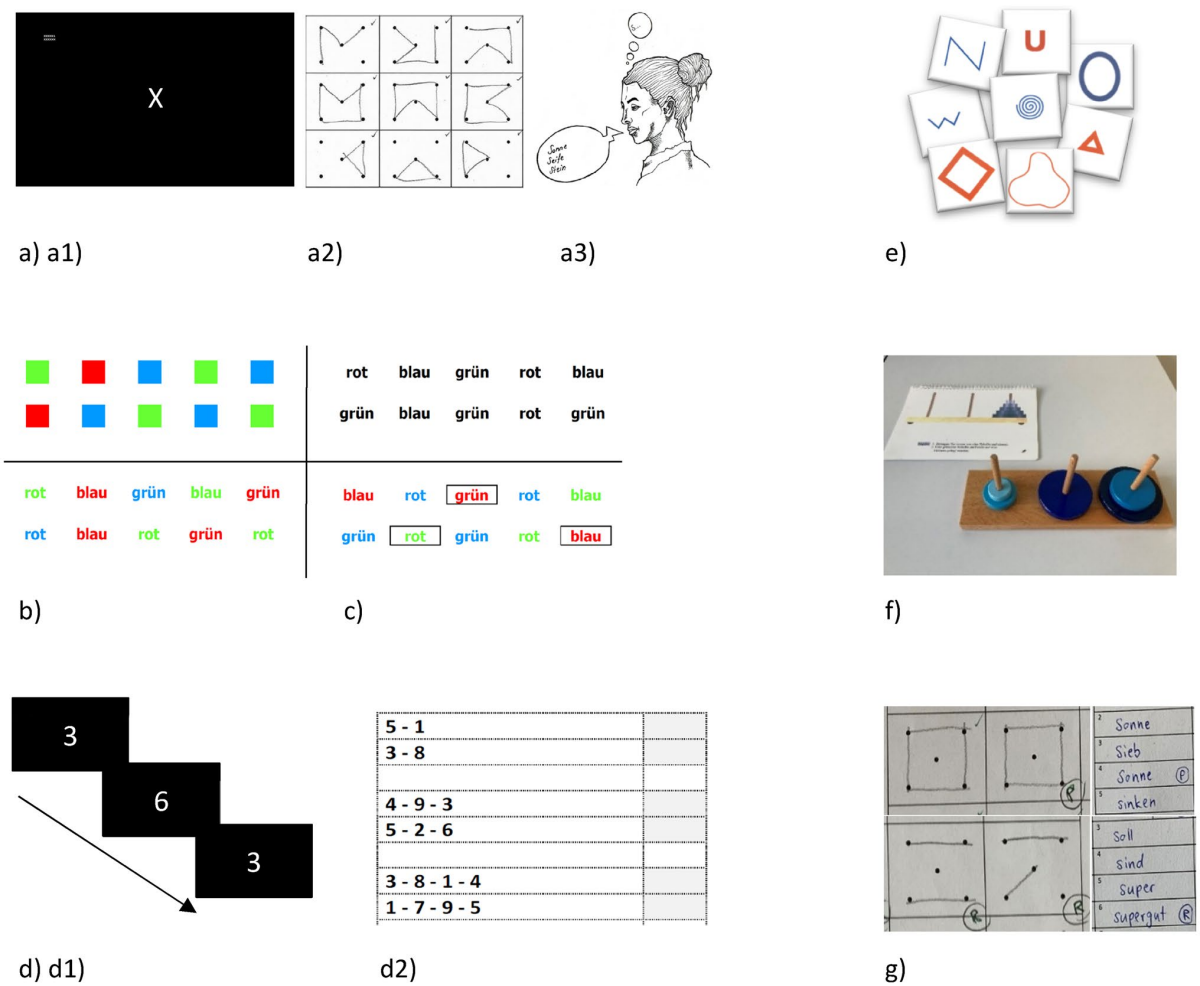
Neuropsychological tests to assess basic and complex executive functions. Basic and complex executive functions were measured with validated neuropsychological tests. Basic executive functions (on the left) were measured with the following tests: **a** *initiation*: **a1** *general initiation* with the Alertness test, **a2** *nonverbal initiation* with the Design Fluency test, **a3** *verbal initiation* with the Word Fluency test; **b** *inhibition* with the Inhibition condition of the Color–Word Interference test; **c** *cognitive flexibility* with the Switching condition of the Color–Word Interference test, **d** *working memory*: **d1** *performance* with the 2-back task, **d2** *maximal span* with the digit span backwards test. Complex executive functions (on the right) were measured with the following tests: **e** *problem solving* with the two groups test; **f** *planning* with the Tower of London; **g** *monitoring* by computing a mean score of errors, rule violations, and perseverations of the other neuropsychological tests. See appendix for detailed task descriptions and references

We comprehensively measured executive functions with several validated neuropsychological tests. Our test battery included basic executive functions (initiation, inhibition, cognitive flexibility, working memory) and complex ones (problem solving, planning, monitoring). An overview of the executive tests is shown in Table 1 and illustrated in Fig. 1. Executive tests were nonspatial if possible. We chose tests that are solvable despite hearing loss, and we prepared written instructions for participants suffering from hearing loss. We added one subjective measure of executive impairments (Frontal System Behaviour Scale, [40]). Beside executive functions, we measured the impact of PVD on daily life functioning (Neuropsychological Vertigo Inventory, [55]). As control variables, we assessed intelligence and global cognitive level. The neuropsychological tests used in this study are described in detail in the appendix.

**Table 1:** Overview of the neuropsychological tests and questionnaires to assess executive functions, impact of peripheral vestibular dysfunction on daily life functioning, and control variables (intelligence, global cognitive level)

Executive function/cognitive domain	Subdomain	Test name	Manual	References	Dependent variable	Higher values indicate
Initiation	General	Alertness test	Testbatterie zur Aufmerksamkeitsprüfung	[107]	Median of the reaction times in tonic Alertness	Worse performance
	Nonverbal	Design Fluency test	Materialien und Normwerte für die Neuropsychologische Diagnostik	[7]	Number of correctly drawn patterns	Better performance
	Verbal	Word Fluency test	Materialien und Normwerte für die Neuropsychologische Diagnostik	[7]	Number of correctly mentioned words	Better performance
Inhibition		Color–Word Interference test: Inhibition	Delis–Kaplan Executive Function System	[25]	Contrast score to parcel out *Color Naming* from performance on *Inhibition*	Better performance
Cognitive flexibility		Color–Word Interference test: Switching	Delis–Kaplan Executive Function System	[25]	Contrast score to parcel out *Color Naming* and *Word Reading* from performance on *Switching*	Better performance
Working memory	Performance	2-back task	Testbatterie zur Aufmerksamkeitsprüfung	[107]	Number of omissions	Worse performance
	Span	Digit span backwards test (visual version)	Wechsler's Memory Scale—Revised	[43]	Sequence of maximal digits correctly solved	Better performance
Problem solving		Two groups test	Materialien und Normwerte für die Neuropsychologische Diagnostik	[7]	Number of correctly mentioned features	Better performance
Planning		Tower of London	Delis–Kaplan Executive Function System	[25]	Total achievement score (correctly built towers within time limit and minimum number of moves possible)	Better performance
Monitoring		Computed score from the other neuropsychological tests	–	–	Mean score of errors, rule violations, and perseverations	Worse performance
Subjective executive functions		Self-report form of the Frontal System Behaviour Scale (FrSBe)	Frontal System Behaviour Scale	[40]	Sum of the frequency values of all questions	More severe subjective executive impairments
Impact of PVD on daily life functioning		Neuropsychological vertigo inventory (NVI)	Neuropsychological Vertigo Inventory	[55]	Sum of the frequency values of all questions	More severe neuropsychological complaints
Intelligence		Matrices test	Wechsler Adult Intelligence Scale—Fourth Edition	[102]	Number of correctly chosen patterns	Higher intelligence
Global cognitive level		Montreal Cognitive Assessment (MoCA)	Montreal Cognitive Assessment	[72]	Overall score	Better global cognitive level

The neuropsychological tests are described in detail in the appendix. Dependent variables were chosen according to the respective test manuals

*PVD* peripheral vestibular dysfunction

### Data analysis

We used double data entry approaches for neuropsychological evaluations and data entry. Two independent examiners evaluated the neuropsychological data by judging or summing up scores. Two independent examiners transformed paper–pencil to digital values. Using double data entry approaches, we avoided both rater subjectivity and transmission errors. We used raw values from the neuropsychological tests to compare performance of patients with PVD to controls. By recruiting pairwise matched healthy controls, we can rule out influences of sex, age, and education on interpretations of neuropsychological raw values.

Statistical analyses were performed in R (version 4.3.1, [76]). First, it was analyzed whether patient groups differ significantly from their respective control group in control variables of intelligence and global cognitive level. We verified normal distributions with Shapiro–Wilk tests. We used two-sided paired *t*-tests to examine whether mean performance of intelligence and global cognitive level in patient groups significantly differed from their respective control group.

Second, we analyzed whether PVD patient groups differed in executive performance from their respective control group. We verified normal distributions with Shapiro–Wilk tests. Most of the tests were distributed normally. Therefore, we used two-sided paired *t*-tests for the executive tests to examine whether mean performance in patient groups significantly differed from their respective control group. To correct for alpha inflation error because of multiple comparisons, we used Bonferroni–Holm corrections. We report uncorrected as well as alpha inflation corrected results.

Third, we exploratively analyzed whether non-vestibular related variables in patients and controls (hearing loss, affective disorders) explain differences in executive performance between patients and controls. We computed linear regression models for executive tests that differed significantly between patients and controls with the covariates hearing loss and affective disorders.

Fourth, we exploratively analyzed whether vestibular related variables in patients (disease duration, symptoms, dizziness handicap, deafferentation degree, and compensation) predict worse executive performance. We computed multiple regression analysis for the executive tests that differed significantly between patients and controls with the vestibular related variables as covariates. Two covariates (symptoms and dizziness handicap) showed high multicollinearity (*R* > 0.70, *p* < 0.001). Therefore, we took the mean of those variables into the multiple regression models.

## Results

### Data exclusion

Fifteen patients had to be excluded due to recovered vestibular function (*n* = 9), exclusion criteria that the patients did not disclose prior to study participation (*n* = 4), or incidental findings in magnetic resonance imaging (MRI) that was assessed for another study (*n* = 2). Therefore, we analyzed data from 83 patients whereof 34 had bilateral, 29 chronic unilateral (right-sided *n* = 15, left-sided *n* = 14), and 20 acute unilateral (right-sided *n* = 12, left-sided *n* = 8) PVD. PVD had different etiologies. In patients with bilateral PVD, the etiologies were: idiopathic on both sides (*n* = 16), meningitis (*n* = 8), vestibular schwannoma and idiopathic on the contralateral side (*n* = 3), vestibular schwannoma and vestibular neuritis on the contralateral side (*n* = 1), endolymphatic hydrops on both sides (*n* = 3), vestibular neuritis and idiopathic on the contralateral side (*n* = 1), vestibular neuritis on both sides (*n* = 1), and gentamycin ototoxicity (*n* = 1). In patients with chronic unilateral PVD, the etiologies were: vestibular schwannoma (*n* = 18), vestibular neurectomy (*n* = 3), labyrinthectomy (*n* = 1), endolymphatic hydrops (*n* = 3), vestibular neuritis (*n* = 2), Zoster oticus (*n* = 1), and meningitis (*n* = 1). In patients with acute unilateral PVD, the etiologies were: vestibular neuritis (*n* = 18), and idiopathic (*n* = 2). Demographic and clinical data of the patients are provided in Tables 2 and 3. Detailed clinical data of individual patients (diagnosis, semicircular function, otolithic function, and deafferentation degree) are presented in the Supplementary Material (Supplementary Table S2).

**Table 2:** Demographic data of the patient groups with different conditions of peripheral vestibular dysfunction (bilateral, chronic unilateral, acute unilateral) and their respective healthy controls

	Bilateral PVD vs. bilateral controls		Chronic unilateral PVD vs. chronic unilateral controls		Acute unilateral PVD vs. acute unilateral controls	
	Bilateral PVD (*n* = 34)	Bilateral controls (*n* = 34)	Chronic unilateral PVD (*n* = 29)	Chronic unilateral controls (*n* = 29)	Acute unilateral PVD (*n* = 20)	Acute unilateral controls (*n* = 20)
Sex (female/male)	14/20	14/20	12/17	12/17	6/14	6/14
Age (years)^a^	51.42 ± 16.42	51.90 ± 16.23	57.98 ± 11.02	58.23 ± 12.12	49.12 ± 15.67	49.09 ± 16.61
Education^a,b^	2.71 ± 1.66	2.94 ± 1.56	2.90 ± 1.57	2.93 ± 1.46	3.85 ± 1.50	3.45 ± 1.64
Intelligence^a,c^	15.91 ± 5.29	17.47 ± 3.96	17.31 ± 4.74	16.52 ± 3.90	17.15 ± 4.61	16.70 ± 4.21
Global cognitive level^a,d^	25.77 ± 2.96	26.60 ± 2.18	26.62 ± 2.37	26.93 ± 2.27	26.10 ± 2.47	26.55 ± 2.61
Hearing loss^e^ (yes/no)	19/15*	1/33*	24/5*	1/28*	3/17	0/20
Depression^e^	3	1	2	0	2	0
Anxiety^e^	2	0	2	0	1	0

*PVD* peripheral vestibular dysfunction

^a^Results are presented as mean ± standard deviation

^b^Education measured as maximal education reached. maximal value = 7

^c^Intelligence assessed with Matrices test (Wechsler Adult Intelligence Scale)

^d^Global cognitive level assessed with Montreal Cognitive Assessment. maximal value = 30

^e^Hearing loss, depression, and anxiety disorders measured by self-ratings

*Asterisks indicate significant differences between patient groups and their respective control group (*p* < 0.001)

**Table 3:** Clinical data of the patient groups with different conditions of peripheral vestibular dysfunction (bilateral, chronic unilateral, acute unilateral)

	Bilateral (*n* = 34)	Chronic unilateral (*n* = 29)	Acute unilateral (*n* = 20)
Lesion side (right/left)	–	15/14	12/8
Disease duration^a^	19.30 ± 17.13 years	10.02 ± 7.00 years	22.00 ± 5.50 days
Symptoms^a,b^	26.60 ± 15.86	20.31 ± 15.36	21.60 ± 20.05
Dizziness handicap^a,c^	28.24 ± 19.06	27.55 ± 19.47	38.13 ± 29.00
Vestibular deafferentation degree^d^ (complete/incomplete)	13/21	12/17	3/17
Vestibular compensation^a,e^	1.59 ± 1.15	1.39 ± 1.39	1.94 ± 1.31

^a^Results are presented as mean ± standard deviation

^b^Symptoms measured with 10-point Likert scales about the intensity of vestibular symptoms. maximal value = 70

^c^Dizziness handicap measured with the Dizziness Handicap Inventory. maximal value = 100

^d^Vestibular deafferentation degree: complete = pathological semicircular canal and otolithic functions. incomplete = pathological semicircular canal function (caloric test), but intact otolithic functions

^e^Vestibular compensation evaluated by the degree of body sway on the dynamic posturography during the task walking in place eyes closed (Unterberger testing). Values close to zero indicate less body sway and better vestibular compensation

Five controls had to be excluded due to exclusion criteria that the controls did not disclose prior to study participation (*n* = 2) or incidental findings in MRI that was assessed for another study (*n* = 3). Therefore, we analyzed data from 49 healthy controls. Demographic data of the controls are provided in Table 2.

For some neuropsychological tasks, we had to exclude patients and their paired healthy controls due to missing values of patients or their respective controls. We had missing values in *verbal initiation* (1 bilateral), *inhibition* and *cognitive flexibility* (1 bilateral, 1 acute unilateral), *working memory performance* (3 bilateral, 2 chronic unilateral, 3 acute unilateral), and *global cognitive level* (4 bilateral). Some questionnaires about *subjective executive functions* and *impact of PVD on daily life functioning* were not returned by patients or their respective healthy control (5 bilateral, 2 chronic unilateral, 1 acute unilateral).

### Control variables

Means and standard deviations of the two control variables (*intelligence, global cognitive level*) are shown in Table 2. *Intelligence* did not differ significantly between patients with bilateral PVD and respective controls (*t*(33) = − 1.78, *p* = 0.084), patients with chronic unilateral PVD and respective controls (*t*(28) = 0.68, *p* = 0.501), or patients with acute unilateral PVD and respective controls (*t*(19) = 0.37, *p* = 0.715). Similarly, *global cognitive level* did not differ significantly between patients with bilateral PVD and respective controls (*t*(29) = − 1.54, *p* = 0.134), patients with chronic unilateral PVD and respective controls (*t*(28) = − 0.52, *p* = 0.610), or patients with acute unilateral PVD and respective controls (*t*(19) = − 0.46, *p* = 0.648).

### Executive functions

Means and standard deviations of all executive tests from patients with bilateral, chronic unilateral, and acute unilateral PVD and their respective controls are shown in Table 4. To compare executive performance between patient groups and their respective control groups, t-values, p-values, and p-values corrected for multiple comparisons are shown in Table 4.

**Table 4:** Executive performance of the patient groups with different conditions of peripheral vestibular dysfunction (bilateral, chronic unilateral, acute unilateral) and their respective healthy controls

	Bilateral PVD vs. bilateral controls					Chronic unilateral PVD vs. chronic unilateral controls					Acute unilateral PVD vs. acute unilateral controls				
	Bilateral (*n* = 34)	Controls (*n* = 34)	*t*	*p*	p*	Chronic unilateral (*n* = 29)	Controls (*n* = 29)	*t*	*p*	p*	Acute unilateral (*n* = 20)	Controls (*n* = 20)	*t*	*p*	p*
Initiation^a^															
General	277.35 ± 61.70	274.47 ± 108.47	0.13	0.893	1.000	283.03 ± 75.67	278.48 ± 115.24	0.16	0.873	1.000	318.10 ± 208.90	283.50 ± 137.82	0.62	0.541	1.000
Nonverbal	26.12 ± 8.57	29.15 ± 8.38	− 1.69	0.101	1.000	28.14 ± 6.37	28.86 ± 7.60	− 0.53	0.597	1.000	31.20 ± 9.52	32.65 ± 8.12	− 0.81	0.430	1.000
Verbal	**21.48 ± 7.81	**28.55 ± 8.71	− 4.45	< 0.001	< 0.001	25.17 ± 7.96	28.86 ± 8.31	− 1.64	0.112	1.000	*24.65 ± 5.79	*28.75 ± 8.12	− 2.10	0.049	0.497
Inhibition^a^	12.12 ± 1.58	11.70 ± 1.70	0.92	0.364	1.000	11.17 ± 1.95	11.66 ± 1.88	− 0.92	0.365	1.000	11.84 ± 2.52	11.58 ± 1.35	0.50	0.621	1.000
Cognitive flexibility^a^	11.00 ± 2.44	10.55 ± 1.86	0.78	0.438	1.000	10.55 ± 2.53	11.00 ± 1.75	− 0.80	0.431	1.000	11.11 ± 2.28	10.32 ± 1.67	1.31	0.208	1.000
Working memory^a^															
Performance	2.87 ± 3.23	2.81 ± 2.65	0.09	0.929	1.000	2.70 ± 2.74	3.15 ± 2.93	− 0.58	0.570	1.000	1.88 ± 1.76	2.12 ± 2.37	− 0.33	0.746	1.000
Maximal span	**4.35 ± 1.20	**5.44 ± 1.31	− 3.67	< 0.001	0.009	4.93 ± 1.25	5.21 ± 1.26	− 0.79	0.438	1.000	*4.90 ± 1.07	*5.80 ± 1.11	− 2.78	0.012	0.119
Problem solving^a^	4.00 ± 1.26	4.44 ± 1.26	− 1.63	0.113	1.000	4.21 ± 1.35	4.48 ± 1.09	− 1.05	0.302	1.000	4.55 ± 1.23	4.60 ± 1.35	− 0.12	0.908	1.000
Planning^a^	20.32 ± 5.20	19.76 ± 4.81	0.48	0.631	1.000	19.41 ± 5.87	19.79 ± 4.51	− 0.29	0.772	1.000	18.80 ± 4.03	19.50 ± 4.61	− 0.51	0.618	1.000
Monitoring^a^	2.21 ± 2.22	1.69 ± 1.31	1.40	0.171	1.000	2.14 ± 1.44	2.00 ± 1.28	0.38	0.705	1.000	1.46 ± 1.18	1.73 ± 1.43	− 0.92	0.369	1.000
FrSBe^a^	90.90 ± 22.72	83.60 ± 19.60	1.18	0.248	1.000	78.85 ± 18.11	83.72 ± 16.37	− 1.00	0.329	1.000	84.11 ± 22.42	83.18 ± 20.80	0.11	0.910	1.000
NVI^a^	59.74 ± 15.09	53.40 ± 11.97	1.92	0.065	0.651	60.33 ± 15.42	54.24 ± 11.70	1.64	0.113	1.000	58.58 ± 17.62	53.03 ± 12.51	0.97	0.343	1.000

Performance of executive functions was measured with validated neuropsychological tests (see appendix, Table 1, or Fig. 1)

*PVD* peripheral vestibular dysfunction, *FrSBe* Frontal System Behaviour Scale, *NVI* Neuropsychological Vertigo Inventory

p* *p* values after correcting for multiple comparisons (Bonferroni)

^a^Results are presented as mean ± standard deviation

*Asterisks indicate significant differences between patient groups and their respective control group before correcting for multiple comparisons (*p < 0.050; **p < 0.001)

**Fig. 2:**
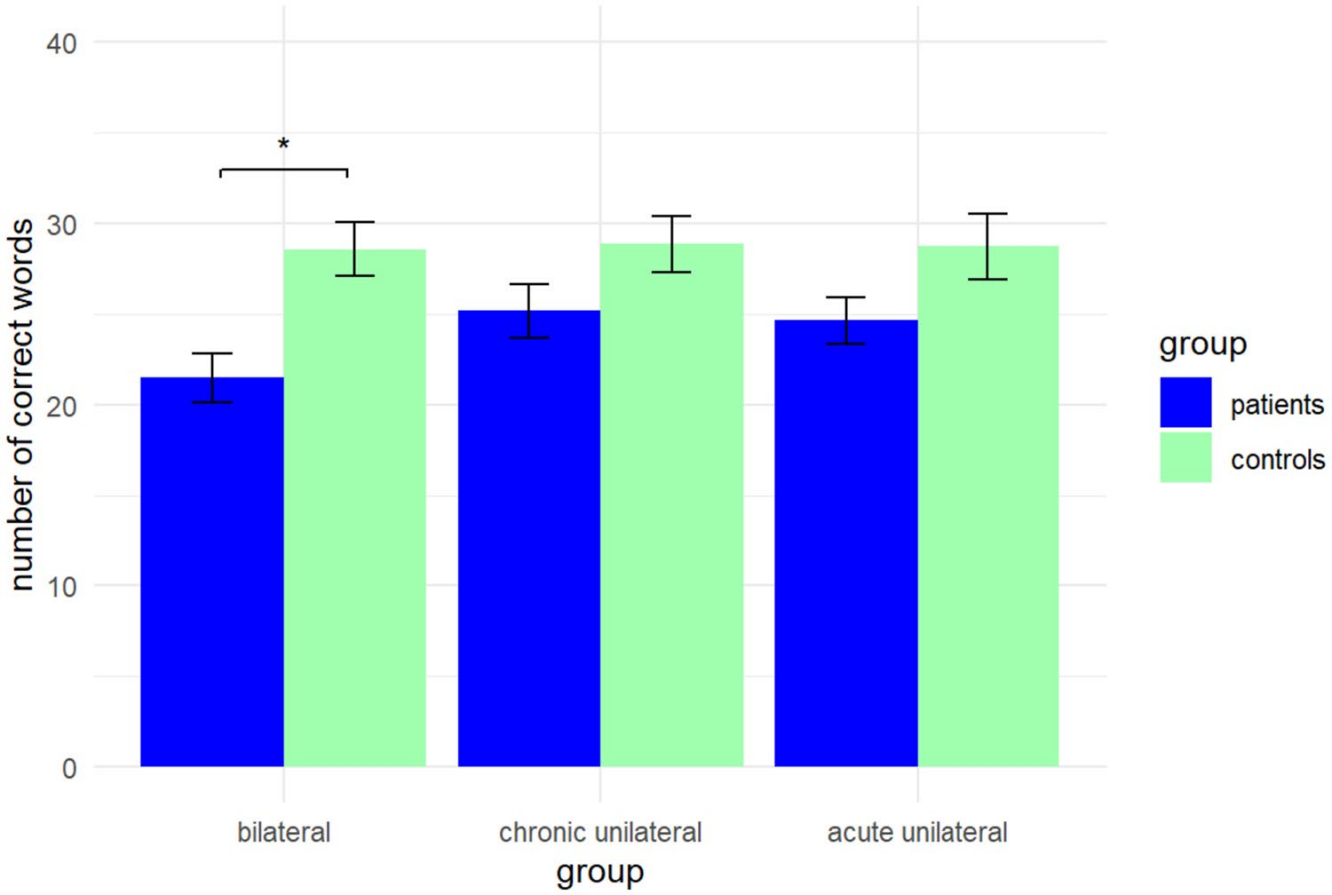
Verbal initiation performance of patients with bilateral, chronic unilateral, and acute unilateral peripheral vestibular dysfunction and their respective pairwise matched healthy controls. Verbal initiation was assessed with a word fluency task. Results are presented as means. Error bars show standard errors. Significant differences between patients and controls are indicated with asterisks (*p < 0.001)

*Verbal initiation* differed significantly between patients with bilateral PVD and respective controls (*t*(32) = − 4.45, *p* < 0.001, *d* = 0.77), and between patients with acute unilateral PVD and respective controls (*t*(19) = − 2.10, *p* = 0.049, *d* = 0.47). After Bonferroni correction for multiple comparisons, verbal initiation still significantly differed between patients with bilateral PVD and respective controls (*t*(32) = − 4.45, *p* < 0.001, *d* = 0.77). But patients with acute unilateral PVD did no longer significantly differ in verbal initiation from respective controls (*t*(19) = − 2.10, *p* = 0.497). Patients with chronic unilateral PVD did not differ in verbal initiation from respective controls (*t*(28) = − 1.64, *p* = 0.112). Results of verbal initiation are shown in Fig. 2.

**Fig. 3:**
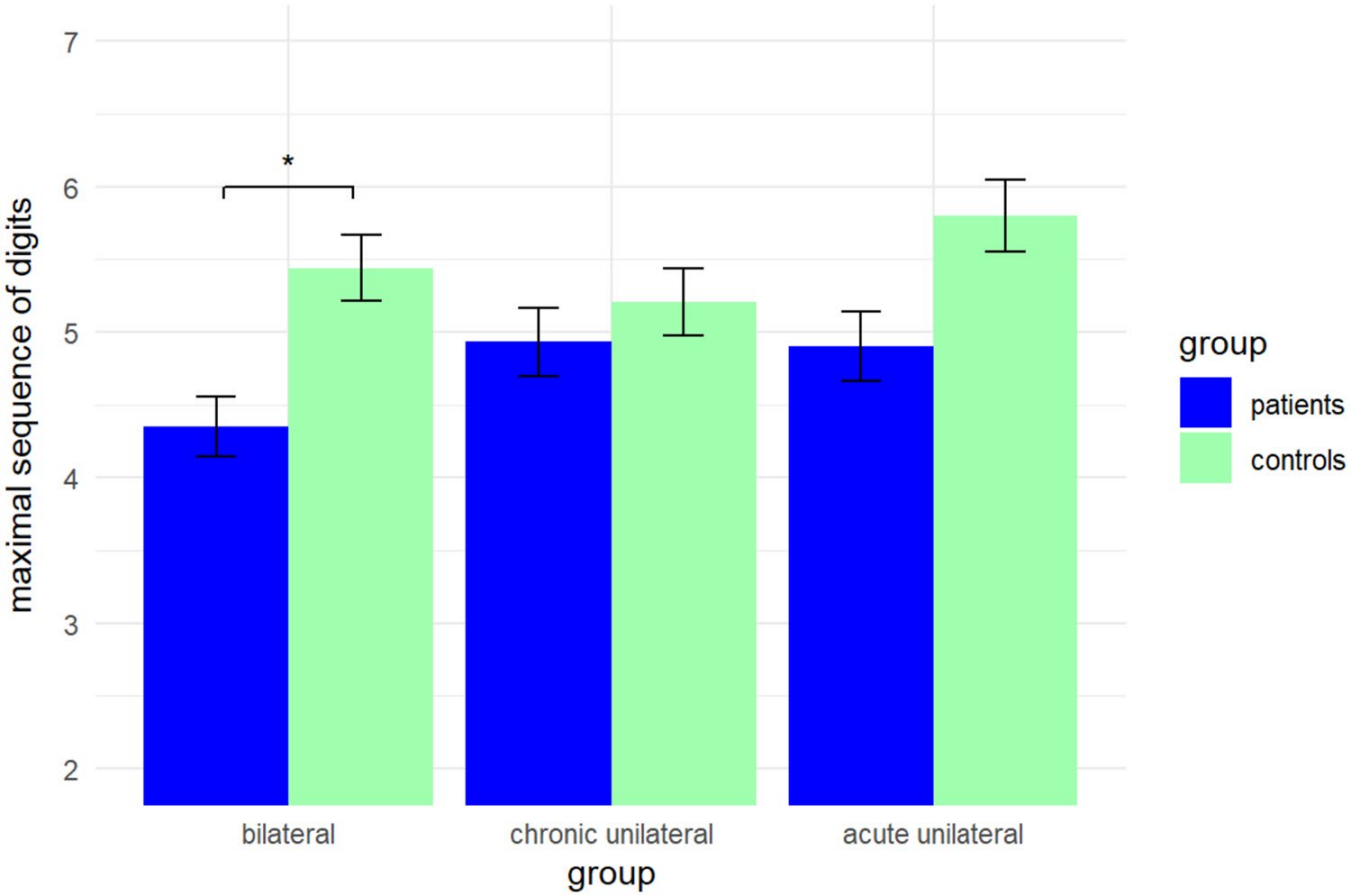
Working memory span of patients with bilateral, chronic unilateral, and acute unilateral peripheral vestibular dysfunction and their respective pairwise matched healthy controls. Working memory span was assessed with a digit span backwards task. Results are presented as means. Error bars show standard errors. Significant differences between patients and controls are indicated with asterisks (*p < 0.001)

Similarly, *working memory span* differed significantly between patients with bilateral PVD and respective controls (*t*(33) = − 3.67, *p* < 0.001, *d* = 0.63), and between patients with acute unilateral PVD and respective controls (*t*(19) = − 2.78, *p* = 0.012, *d* = 0.62). After Bonferroni correction for multiple comparisons, working memory span still significantly differed between patients with bilateral PVD and respective controls (*t*(33) = − 3.67, *p* = 0.009, *d* = 0.63). But patients with acute unilateral PVD did no longer significantly differ in working memory span from respective controls (*t*(19) = − 2.78, *p* = 0.119). Patients with chronic unilateral PVD did not differ in working memory span from respective controls (*t*(28) = − 0.79, *p* = 0.438). Results of working memory span are shown in Fig. 3.

Performance in other executive tests did not differ significantly between patients with bilateral PVD and respective controls, patients with chronic unilateral PVD and respective controls, or patients with acute unilateral PVD and respective controls (*p* > 0.050).

### Non-vestibular related variables and vestibular related variables

Numbers of participants with hearing loss and affective disorders are shown in Table 2. Numbers of participants with complete or incomplete vestibular deafferentation, as well as means and standard deviations of disease duration, symptoms, dizziness handicap, and vestibular compensation are shown in Table 3.

After adjusting for *hearing loss*, verbal initiation and working memory span differed statistically significant between patients with bilateral PVD and respective controls (*F*(3, 63) = 5.00, *p* = 0.004, *d* = 0.35), (*F*(3, 64) = 6.25, *p* < 0.001, *d* = 0.37). After adjusting for *affective disorders*, verbal initiation and working memory span differed statistically significant between patients with bilateral PVD and respective controls (*F*(3, 62) = 4.01, *p* = 0.011, *d* = 0.57), (*F*(3, 63) = 6.05, *p* = 0.001, *d* = 0.66).

Vestibular related variables did not predict verbal initiation performance in patients with bilateral PVD. The multiple regression model with verbal initiation as dependent variable and the vestibular related variables as covariates (disease duration, mean of symptoms and dizziness handicap, deafferentation degree, and compensation) was not significant (*F*(4, 27) = 2.20, *p* = 0.096). None of the vestibular related variables significantly predicted verbal initiation (*p* > 0.050).

Vestibular related variables did not predict working memory span in patients with bilateral PVD. The multiple regression model with working memory span as dependent variable and the vestibular related variables as covariates (disease duration, mean of symptoms and dizziness handicap, deafferentation degree, and compensation) was not significant (*F*(4, 28) = 1.26, *p* = 0.308). None of the vestibular related variables significantly predicted working memory span (*p* > 0.050).

## Discussion

We investigated whether executive performance in a large sample of patients with several conditions of PVD (bilateral, chronic unilateral, acute unilateral) differs from pairwise matched healthy controls. Verbal initiation and working memory span were impaired in patients with bilateral and patients with acute unilateral PVD, but not in patients with chronic unilateral PVD. After correcting for multiple comparisons only patients with bilateral PVD performed worse than controls in verbal initiation and working memory span. Performance in all other executive tests (general initiation, nonverbal initiation, inhibition, cognitive flexibility, working memory performance, problem solving, planning, and monitoring) was not impaired in patients with bilateral, chronic unilateral, or acute unilateral PVD compared to their respective controls. We also investigated whether non-vestibular related variables in patients and controls (hearing loss, affective disorders) and vestibular related variables in patients (disease duration, mean of symptoms and dizziness handicap, deafferentation degree, and compensation) influenced our results. Hearing loss and affective disorders did not explain differences between patients with bilateral PVD and controls. None of the vestibular related variables predicted verbal initiation or working memory span in patients with bilateral PVD.

### Executive impairments in patients with bilateral PVD

Impaired verbal initiation in patients with bilateral PVD conflicts with a previous result showing normal verbal initiation in patients with bilateral PVD [101]. Two differences can account for the diverging results. First, Wang et al. [101] used a semantic fluency task to assess verbal initiation, whereas we used a word fluency task. These tasks might not be comparable and assess different aspects of verbal initiation [9, 60, 80]. Second, Wang et al.'s [101] sample included patients with only mild bilateral PVD, whereas we included patients with complete vestibular deafferentation. More severe cognitive deficits are expected in patients with complete vestibular deafferentation [38].

Impaired working memory span in patients with bilateral PVD confirms results from a previous study [23]. Using the same task, Danneels et al. [23] found impaired working memory span in patients with bilateral PVD with and without hearing loss. The mean working memory span of our patients with bilateral PVD (*M* = 4.35) is highly comparable to the bilateral patient group with hearing loss in the study of Danneels et al. (*M* = 4.37) [23]. However, our results conflict with normal working memory span in patients with bilateral PVD, which was also assessed with the same task [1]. Contrary to our study, Ahmad et al. [1] excluded patients with surgery, tumors, neurectomy, ototoxic medication, and hearing loss. Consequently, it is likely that patients with bilateral PVD in the study of Ahmad et al. [1] had a lower degree of vestibular deafferentation. Impaired working memory span may show up only in patients with a high degree of vestibular deafferentation [38]. In addition, Ahmad et al. [1] based diagnosis of bilateral PVD exclusively on rotary chair testing, and they did not assess normal vestibular function in healthy controls. Potential mild PVD in some healthy controls might have biased their results. Van Hecke et al. [98] found normal working memory span in children with bilateral PVD. However, compared to healthy controls, average performance of children with bilateral PVD was reduced. The small sample size (*n* = 9) might not have allowed enough statistical power to detect differences between children with bilateral PVD and controls.

Although patients with bilateral PVD performed worse in working memory span (digit span backwards), they performed normally in working memory performance (2-back task). Working memory span and working memory performance correlate weakly and seem to measure different aspects of working memory [50, 96]. In a 2-back task that we used in our study, the maximal span to be held in working memory is two. Patients may not have problems with working memory tasks limited to a span of two but are impaired in reaching longer spans. However, n-back tasks have been criticized in that they are insufficient reliable and do not purely measure working memory but rely more on information processing or motor speed [49, 50, 67]. Moreover, it has been suggested that n-back tasks are not a useful measure of individual differences in working memory [49]. Therefore, results from the digit span backwards task may be more reliable. The digit span backwards task is also assumed to be a representative measure of working memory [27, 45].

Considering our comprehensive assessment of executive functions, we argue that patients with bilateral PVD suffer from a specific working memory span impairment rather than general executive impairments. Specifically impaired working memory span, but preserved other executive functions have also been observed in healthy participants receiving high-current galvanic vestibular stimulation [81]. Patients with bilateral PVD performed equally as healthy controls in all other assessed executive components. In fact, the impaired verbal initiation that we also observed in these patients may be explained by working memory span problems, because verbal fluency tasks rely on working memory capacity [6, 30, 33, 66].

A possible mechanism between bilateral PVD and impaired working memory span may be structural changes in or disturbed vestibular input to the inferior frontal gyrus. The inferior frontal gyrus is involved in vestibular processing [61, 68]. Gray matter volume increases in this area have been associated with vestibular compensation after vestibular neuritis [48]. Interestingly, the inferior frontal gyrus is also activated during working memory tasks and verbal fluency tasks [46, 73, 103]. Previous studies in patients with bilateral PVD reported structural changes predominantly in the hippocampus or hippocampal substructures following PVD [15, 38, 52, 82]. Future studies should use structural MRI to examine alterations in the inferior frontal gyrus in patients with bilateral PVD. In addition, the inferior frontal gyrus in patients with bilateral PVD should be investigated in functional imaging studies while patients are solving a working memory or verbal fluency task in the MRI scanner.

Alternatively, results could be explained by a speech problem in patients with bilateral PVD. Verbal initiation and working memory span were the only tasks in our test battery that required actively produced speech-based responses. However, we think that this explanation is unlikely. Previous studies showed no general speech problem in patients with bilateral PVD [13]. Moreover, previous studies reporting visuo-spatial working memory impairments did not use speech-based tasks [56, 75]. Nevertheless, future studies should use also non-speech-based working memory tasks to exclude an influence of speech.

We are able to exclude some alternative explanations for the impaired verbal initiation and working memory span in patients with bilateral PVD. First, contrary to some earlier studies [26, 31, 56], we used nonspatial tasks and can therefore rule out visuo-spatial influences on executive performance. The working memory span task may have a visuo-spatial component (mental number line), but the verbal initiation task does not rely on visuo-spatial performance. In addition, patients with bilateral PVD did not differ from healthy controls in other tasks using numbers in our test battery. Second, our results in patients with bilateral PVD remained significant after controlling for hearing loss or affective disorders. This confirms recent results showing that PVD is associated with cognitive dysfunction independent of hearing loss [86]. Third, variables such as disease duration, symptoms, dizziness handicap, deafferentation degree, or compensation did not predict verbal initiation performance or working memory span. However, the influence of vestibular related variables on executive performance was not the main aim of our study and we did not recruit patients respectively. Future studies should investigate executive performance in combination with vestibular related variables (e.g., compare patients with symptoms vs. no symptoms or patients with complete deafferentation vs. incomplete deafferentation). Fourth, impaired verbal initiation and working memory span in patients with bilateral PVD cannot be explained by impaired intelligence or global cognitive level, as those variables did not differ from those of healthy controls.

### No executive impairments in patients with acute unilateral PVD?

After controlling for multiple comparisons, the effect of impaired verbal initiation and working memory span in patients with acute PVD diminished, although the exact same pattern of impairments as in patients with bilateral PVD has been observed descriptively. Patients with acute unilateral PVD might suffer from impaired verbal initiation and working memory span, but the effects are less strong when compared to patients with bilateral PVD. Nonsignificant results after correcting for multiple comparisons could be explained by a smaller sample size of patients with acute PVD (*n* = 20) compared to patients with bilateral PVD (*n* = 34). We assume that patients with acute PVD suffer from impaired verbal initiation and working memory span, but we were not able to demonstrate the effect with our data. Future studies should examine this line of research further.

### No executive impairments in patients with chronic unilateral PVD

Patients with chronic unilateral PVD did not perform worse in executive tasks than healthy controls. This finding confirms some previous results [1, 75]. However, it conflicts results of previously observed impaired executive functions in patients with chronic unilateral PVD [26, 31, 56, 75, 77]. Conflicting results are most likely due to the fact that earlier studies used visuo-spatial measures [26, 31, 56] or did not control for processing speed when assessing executive functions [31, 77]. Fan et al. [31] found impaired working memory in patients with chronic unilateral PVD using a nonspatial task. However, their sample included exclusively patients with untreated acoustic neurinoma, whereas our sample included treated and untreated patients. Interestingly, Popp et al. [75] used the same task to assess general initiation that was used in our study. However, contrary to our study, Popp et al. [75] did not adapt the task for hearing loss, although this is suggested in a vestibular patient group [28].

We can rule out that normal executive performance in patients with chronic unilateral PVD is due to less symptoms or dizziness handicap than in patients with bilateral or patients with acute unilateral PVD. Patients with chronic unilateral PVD reported nearly equal intensity of symptoms as patients with acute PVD and dizziness handicap as patients with bilateral PVD. However, patients with chronic unilateral PVD had better vestibular compensation values than patients with bilateral or patients with acute unilateral PVD. Although this difference was not statistically significant, we argue that vestibular compensation can prevent executive problems. Acute unilateral PVD may lead to impaired working memory span, but vestibular compensation can help to recover from those impairments. Future longitudinal studies with patients with acute unilateral PVD should examine this line of research.

### Implications

As patients with PVD are treated by various multidisciplinary work groups [79], the knowledge gained from our results are not only important for neurotologists, but also for neurologists, audiologists, or other patient support groups. Improving diagnostic and treatment procedures for patients with PVD requires awareness of cognitive impairments in those patients.

Reduced working memory span can have intense negative consequences, because intact working memory is crucial for everyday life, at the workplace, or in social situations (e.g., [16–18, 27, 36, 59, 78]). Therefore, impaired working memory span might lead to those deficiencies in daily life activities that have been reported in patients with PVD [44]. Compared to other executive functions, working memory best predicted impairments in daily life in older adults [99]. Moreover, working memory predicts academic performance (e.g., [24, 85, 100]).

Due to its wide-ranging negative consequences, impaired working memory span in patients with bilateral PVD should be treated. We suggest interventions to treat impaired working memory that have been investigated in other patient populations or healthy participants. These intervention suggestions should be investigated in patients with bilateral PVD in future studies.

The most obvious strategy to treat impaired working memory would be a cognitive training in the form of a working memory training. In such a training, participants learn, for example, to improve their working memory span by repeated sessions of a backward memory span task (e.g., [58, 106]). It has been shown that effects of a working memory training transfer to other tasks not specifically trained [16, 17, 58, 78, 106]. Therefore, such a training could improve a variety of tasks in daily life or at work. It has been shown that executive performance has a predictive effect on balance and gait in older adults or cognitively impaired participants [5, 89–91, 95, 97, 105]. Thus, a working memory training might have additional advantageous side effects on balance functions in patients with PVD.

It has to be pointed out that a cognitive training is demanding and needs a high patient motivation. Alternatively, patients could benefit from psychoeducational offers. Psychoeducation could include concrete suggestions in dealing with impaired working memory span or, for example, altered working strategies or working time adjustments at the workplace. Psychoeducational strategies are generally less demanding for patients. However, positive effects are limited to a specific problem and adaptations at the workplace need the consent of employers.

Integrating screenings for executive impairments (working memory span, verbal initiation) in standard neurotological assessments would help to identify patients with executive problems. This would allow to intervene in an early stage after PVD and prevent long-term consequences that may result from cognitive impairments (e.g., absence from work).

### Strengths and limitations

This study has several strengths. First, we assessed a comprehensive range of executive functions including basic and complex executive functions. We used nonspatial, validated neuropsychological tests and controlled for processing speed when measuring reaction times. Therefore, we can make thorough conclusions about executive impairments ruling out influences of impaired spatial cognition or processing speed. Second, executive tests were administered in a highly standardized manner. In addition, using double data entry approaches for test evaluation and data entry, we avoided rater subjectivity and transmission errors. Third, we recruited pairwise matched healthy control groups highly comparable to patients in sex, age, and education. With our recruitment strategy, we minimized demographic influences on executive performance. Fourth, we integrated a large patient sample and several conditions of PVD (laterality, course). Conclusions about different PVD groups can facilitate strategies in clinical diagnostics and rehabilitation.

Besides its strengths, our study has two limitations. First, although we included patients with different conditions of PVD (bilateral, chronic unilateral, and acute unilateral), patients within the groups had heterogeneous diagnoses. However, the dysfunction of vestibular input is likely more influential on executive performance than the underlying specific diagnosis. Future studies will need to compare different conditions (laterality, course) and also different diagnosis leading to PVD. Second, as this was not the primary objective of the study, we assessed hearing loss and affective disorders by questioning participants instead of using validated questionnaires or objective measures. Therefore, we had no fine-grained assessment of hearing loss or affective disorders, and we could have missed weak expressions. Future studies should assess those variables with validated or objective tests. However, the results of this study can hardly be explained by hearing loss or affective disorders as these conditions would be expected to impair cognition more generally rather than specific executive functions in isolation (e.g., [41], [63]).

## Conclusion

Patients with bilateral peripheral vestibular dysfunction performed worse in the specific executive functions of verbal initiation and working memory span when compared to pairwise matched healthy controls. Patients with bilateral peripheral vestibular dysfunction should be screened for executive impairments and if indicated, receive cognitive training or psychoeducation.

### Electronic supplementary material

Below is the link to the electronic supplementary material.

Supplementary file1 (Pdf 424 KB)

## Data Availability

The data that has been used is confidential.

## Appendix

Detailed descriptions of the neuropsychological tests and questionnaires used in the study.

*Initiation* was measured by three subdomains: general, nonverbal, and verbal.

*General initiation* was measured with the Alertness test (Testbatterie zur Aufmerksamkeitsprüfung, [107]). The Alertness test is a simple reaction time task. Participants must respond as fast as possible by pressing a key when a cross appears on the screen. We used the tonic Alertness task (without warning tone) with 40 trials and assessed the median of the reaction times, according to the manual. Higher values indicate worse performance.

*Nonverbal initiation* was assessed with the Design Fluency test (Materialien und Normwerte für die Neuropsychologische Diagnostik, [7]). Participants must draw as many different patterns as possible by connecting two to five dots within 3 min. They were not allowed drawing curved or disconnected lines. We assessed the number of correctly drawn patterns, according to the manual. Higher values indicate better performance.

*Verbal initiation* was evaluated with the Word Fluency test (Materialien und Normwerte für die Neuropsychologische Diagnostik, [7]). Participants must enumerate as many words as possible beginning with the letter “S” within 3 min. They were not allowed using the same word stem repeatedly and mentioning personal names or place names. We assessed the number of correctly mentioned words, according to the manual. Higher values indicate better performance.

*Inhibition* was measured with the Color–Word Interference test (Delis–Kaplan Executive Function System, [25]). Participants solved three conditions: (1) *Color Naming*: naming colored squares, (2) *Word Reading*: reading black printed color words (e.g., the word “red” printed in black ink), (3) *Inhibition*: naming the ink of incongruent color words, while inhibiting reading the words (e.g., name “blue” for the word “red” printed in blue ink). The first two conditions are control conditions. We computed a contrast score to parcel out *Color Naming* from performance on *Inhibition* to control for motor speed, according to the manual. Higher contrast scores indicate better performance.

*Cognitive flexibility* was assessed with an additional condition of the Color–Word Interference test (Delis–Kaplan Executive Function System, [25]), *Switching*: Participants have to switch between two tasks: naming and reading. They should name the ink of incongruent color words (e.g., name “blue” for the word “red” printed in blue ink, see condition *Inhibition* above) or, if a word is contained in a rectangle, read incongruent color words (e.g., read the word “red” printed in blue ink). We computed a contrast score to parcel out *Color Naming* and *Word Reading* from performance on *Switching*, according to the manual. Higher contrast scores indicate better performance.

*Working memory* was determined by two subdomains: performance and span. These subdomains correlate weakly and seem to measure different aspects of working memory [50].

*Working memory performance* was measured with the 2-back task (Testbatterie zur Aufmerksamkeitsprüfung, [107]). Numbers from one to nine are presented sequentially on a screen and participants must respond to numbers that are identical with the penultimate one. We assessed the number of omissions, according to the manual. Omissions show an impaired control of information flow and are the most important performance parameter of working memory performance [107]. Higher values indicate worse performance.

*Working memory span* was assessed with a visual version of the digit span backwards test (Wechsler's Memory Scale—Revised, [43]). Participants must keep in mind a visual sequence of digits presented sequentially and repeat the sequence backwards orally. The task starts with two digits and increases in difficulty by longer sequences (max. seven digits). We assessed the sequence of maximal digits that could be solved correctly, according to the manual. Higher values indicate better performance.

*Problem solving *was evaluated with the two groups test (Materialien und Normwerte für die Neuropsychologische Diagnostik, [7]). Eight cards with different symbols are presented to participants. Participants must sort the cards into two groups according to a feature that differentiates four cards from the other four. After sorting, the cards are shuffled, and participants have to find a new feature to group the cards into two groups. There are six possible features to sort the cards: color (blue/red), shape (angular/curved), thickness (thick/thin), unity (open/closed), size (large/small), or letter characterization (letters/non-letters). We assessed the number of correctly mentioned features, according to the manual. Higher values indicate better performance.

*Planning* was measured with the Tower of London (Delis–Kaplan Executive Function System, [25]). A wooden board with three vertical pegs and five disks different in size and shades of blue is presented to participants. Participants must move disks from the start configuration to a target configuration with as few moves as possible without violating task rules. Task rules are, not placing a bigger disk on a smaller one, and not removing two disks from the peg at the same time. We computed the total achievement score, representing correctly built towers within the time limit and the minimum numbers of moves possible, according to the manual. Higher values indicate better performance.

*Monitoring* was computed by a score from the other neuropsychological tests because no validated neuropsychological test exists to measure this executive component. We computed a mean score of errors, rule violations, and perseverations [(errors in the Color − Word Interference test + errors in the 2-back task + rule violations and perseverations in the Design Fluency test + rule violations and perseverations in the Word Fluency test + rule violations in the Tower of London)/7]. In case of a missing value in one of the seven variables, the mean score was computed with the remaining variables. Higher values indicate worse performance.

*Subjective executive functions* were quantified with the self-report form of the Frontal System Behaviour Scale (FrSBe, [40]). The 46-item questionnaire measures behaviors associated with frontal lobe damage of the brain. Participants give frequency ratings on a five-point Likert scale. We assessed the sum of the frequency values of all questions, according to the manual. Higher values indicate more severe subjective executive impairments.

*Impact of PVD on daily life functioning* was assessed with the Neuropsychological Vertigo Inventory (NVI, [55]). The Neuropsychological Vertigo Inventory is a 28-item self-report questionnaire that measures physical, cognitive, and emotional complaints. It was developed to assess neuropsychological symptoms in patients with vertigo. Participants give frequency ratings on a five-point Likert scale. We assessed the sum of the frequency values of all questions, according to the manual. Higher values indicate more severe neuropsychological complaints.

*Intelligence* was measured with the Matrices test (Wechsler Adult Intelligence Scale—Fourth Edition, [102]). Participants must choose one out of five patterns that fits into a given sequence of patterns. The Matrices test correlates highly with general intelligence [84]. We assessed the number of correctly chosen patterns, according to the manual. Higher values indicate higher intelligence.

*Global cognitive level* was evaluated with the Montreal Cognitive Assessment (MoCA, [72]). The assessment consists of a 30-point test that includes eight cognitive domains: visuo-spatial/executive, naming, memory, attention, language, abstraction, delayed recall, and orientation. This test is often used as a screening for dementia or mild cognitive impairment [47]. We assessed the overall score, according to the manual. Higher values indicate better global cognitive level.

## References

[1] Ahmad M, Bola L, Boutabla A, King S, Lewis RF, Chari DA (2022). Visuospatial cognitive dysfunction in patients with vestibular loss. Otol Neurotol.

[2] Allum JHJ, Adkin AL, Carpenter MG, Held-Ziolkowska M, Honegger F, Pierchala K (2001). Trunk sway measures of postural stability during clinical balance tests: effects of a unilateral vestibular deficit. Gait Posture.

[3] Anderson P (2002). Assessment and development of Executive Function (EF) during childhood. Child Neuropsychol.

[4] Ayar DA, Kumral E, Celebisoy N (2020). Cognitive functions in acute unilateral vestibular loss. J Neurol.

[5] Azadian E, Majlesi M, Jafarnezhadgero AA (2018). The effect of working memory intervention on the gait patterns of the elderly. J Bodyw Mov Ther.

[6] Baldo JV, Shimamura AP, Delis DC, Kramer J, Kaplan E (2001). Verbal and design fluency in patients with frontal lobe lesions. J Int Neuropsychol Soc.

[7] Balzer C, Berger JM, Caprez G, Gonser A, Gutbrod K & Keller M (2011) Materialen und Normwerte für die neuropsychologische Diagnostik (MNND). Verlag Normdaten

[8] Besnard S, Lopez C, Brandt T, Denise P, Smith PF (2015). Editorial: The Vestibular System in Cognitive and Memory Processes in Mammalians. Front Integr Neurosci.

[9] Biesbroek JM, van Zandvoort MJE, Kappelle LJ, Velthuis BK, Biessels GJ, Postma A (2016). Shared and distinct anatomical correlates of semantic and phonemic fluency revealed by lesion-symptom mapping in patients with ischemic stroke. Brain Struct Funct.

[10] Bigelow RT, Agrawal Y (2015). Vestibular involvement in cognition: visuospatial ability, attention, executive function, and memory. J Vestib Res.

[11] Bigelow RT, Semenov YR, du Lac S, Hoffman HJ, Agrawal Y (2016). Vestibular vertigo and comorbid cognitive and psychiatric impairment: the 2008 National Health Interview Survey. J Neurol Neurosurg Psychiatry.

[12] Black FO, Pesznecker S, Stallings V (2004). Permanent gentamicin vestibulotoxicity. Otol Neurotol.

[13] Bosmans J, Gommeren H, Mertens G, Cras P, Engelborghs S, Van Ombergen A, Vereeck L, Gilles A, Van Rompaey V (2022). Associations of bilateral vestibulopathy with cognition in older adults matched with healthy controls for hearing status. JAMA Otolaryngol.

[14] Brandt T (1999). Vertigo: Its multisensory syndromes.

[15] Brandt T, Schautzer F, Hamilton DA, Brüning R, Markowitsch HJ, Kalla R, Darlington C, Smith P, Strupp M (2005). Vestibular loss causes hippocampal atrophy and impaired spatial memory in humans. Brain.

[16] Brehmer Y, Westerberg H, Bäckman L (2012). Working-memory training in younger and older adults: training gains, transfer, and maintenance. Front Hum Neurosci.

[17] Cantarella A, Borella E, Carretti B, Kliegel M, de Beni R (2017). Benefits in tasks related to everyday life competences after a working memory training in older adults: working memory training and everyday abilities. Int J Geriatr Psychiatry.

[18] Carretti B, Borella E, Zavagnin M, de Beni R (2013). Gains in language comprehension relating to working memory training in healthy older adults: working memory training in older adults. Int J Geriatr Psychiatry.

[19] Chari DA, Madhani A, Sharon JD, Lewis RF (2022). Evidence for cognitive impairment in patients with vestibular disorders. J Neurol.

[20] Cohen HS, Sangi-Haghpeykar H (2011). Walking speed and vestibular disorders in a path integration task. Gait Posture.

[21] Collins A, Koechlin E (2012). Reasoning, learning, and creativity: frontal lobe function and human decision-making. PLoS Biol.

[22] Conrad J, Habs M, Brandt T, Dieterich M (2015). Acute unilateral vestibular failure does not cause spatial hemineglect. PLoS One.

[23] Danneels M, Van Hecke R, Leyssens L, van de Berg R, Dhooge I, Cambier D, Van Rompaey V, Maes L (2023). Association of Bilateral Vestibulopathy With and Without Hearing Loss With Cognitive-Motor Interference. JAMA Otolaryngol.

[24] Davidson C, Shing YL, McKay C, Rafetseder E, Wijeakumar S (2023). The first year in formal schooling improves working memory and academic abilities. Dev Cogn Neurosci.

[25] Delis DC, Kaplan E, Krammer JH (2001) Delis—kaplan executive function system. Psychological corporation, San Antonio

[26] Deng X, Liu L, Li J, Yao H, He S, Guo Z, Sun J, Liu W, Hui X (2022). Brain structural network to investigate the mechanism of cognitive impairment in patients with acoustic neuroma. Front Aging Neurosci.

[27] Diamond A (2013). Executive functions. Annu Rev Psychol.

[28] Dobbels B, Mertens G, Gilles A, Claes A, Moyaert J, van de Berg R, Van de Heyning P, Vanderveken O, Van Rompaey V (2019). Cognitive function in acquired bilateral vestibulopathy: a cross-sectional study on cognition, hearing, and vestibular loss. Front Neurosci.

[29] Drechsler R (2007). Exekutive Funktionen. Z Neuropsychol.

[30] Eichorn N, Marton K, Schwartz RG, Melara RD, Pirutinsky S (2016). Does working memory enhance or interfere with speech fluency in adults who do and do not stutter? Evidence from a dual-task paradigm. J Speech Lang Hear Res.

[31] Fan Z, Fan Z, Li Z, Zhang H, Hu L, Qiu T, Zhu W (2023). Cognitive performance in patients with sporadic vestibular Schwannoma. Neurosurgery.

[32] Ferrè ER, Haggard P (2020). Vestibular cognition: state-of-the-art and future directions. Cogn Neuropsychol.

[33] Fischer-Baum S, Miozzo M, Laiacona M, Capitani E (2016). Perseveration during verbal fluency in traumatic brain injury reflects impairments in working memory. Neuropsychology.

[34] Formeister EJ, Krauter R, Kirk L, Zhu TR (2019). Understanding the Dizziness Handicap Inventory (DHI): a cross sectional analysis of symptom factors that contribute to DHI variance. Otol Neurotol.

[35] Foubert-Samier A, Catheline G, Amieva H, Dilharreguy B, Helmer C, Allard M, Dartigues J-F (2012). Education, occupation, leisure activities, and brain reserve: a population-based study. Neurobiol Aging.

[36] Furley PA, Memmert D (2012). Working memory capacity as controlled attention in tactical decision making. J Sport Exerc Psychol.

[37] Gioia GA, Isquith PK & Guy SC (2001) Assessment of executive function in children with neuropsychological impairments. In: Psychological and developmental assessment. Guilford Press, pp 317–356

[38] Göttlich M, Jandl NM, Sprenger A, Wojak JF, Münte TF, Krämer UM, Helmchen C (2016). Hippocampal gray matter volume in bilateral vestibular failure: Hippocampal Gray Matter Volume in BVF. Hum Brain Mapp.

[39] Grabherr L, Cuffel C, Guyot J-P, Mast FW (2011). Mental transformation abilities in patients with unilateral and bilateral vestibular loss. Exp Brain Res.

[40] Grace J & Malloy P (2001) Frontal Systems Behavior Scale (FrSBe): professional manual. psychological assessment resources

[41] Gualtieri CT, Dexter WM (2008) The frequency of cognitive impairment in patients with anxiety, depression, and bipolar disorder: an unaccounted source of variance in clinical trials. J Clin Psychiatry 10.4088/jcp.v69n0712 18572982

[42] Guidetti G, Guidetti R, Manfredi M, Manfredi M (2019). Vestibular pathology and spatial working memory. Acta Otorhinolaryngol Ital.

[43] Härting C & Wechsler D (Eds) (2000) Wechsler-Gedächtnistest - revidierte Fassung: WMS-R; Manual; deutsche Adaptation der revidierten Fassung der Wechsler Memory scale (1. Aufl). Huber

[44] Harun A, Semenov YR, Agrawal Y (2015). Vestibular function and activities of daily living: analysis of the 1999 to 2004 National Health and Nutrition Examination Surveys. Gerontol Geriatr Med.

[45] Hilbert S, Nakagawa TT, Puci P, Zech A, Bühner M (2015). The digit span backwards task: verbal and visual cognitive strategies in working memory assessment. Eur J Psychol Assess.

[46] Hirshorn EA, Thompson-Schill SL (2006). Role of the left inferior frontal gyrus in covert word retrieval: neural correlates of switching during verbal fluency. Neuropsychologia.

[47] Hobson J (2015). The Montreal Cognitive Assessment (MoCA). Occup Med.

[48] Hong S-K, Kim JH, Kim H-J, Lee H-J (2014). Changes in the gray matter volume during compensation after vestibular neuritis: a longitudinal VBM study. Restor Neurol Neurosci.

[49] Jaeggi SM, Buschkuehl M, Perrig WJ, Meier B (2010). The concurrent validity of the *N*-back task as a working memory measure. Memory.

[50] Kane MJ, Conway ARA, Miura TK, Colflesh GJH (2007). Working memory, attention control, and the n-back task: a question of construct validity. J Exp Psychol Learn Mem Cogn.

[51] Kim J-S, Newman-Toker DE, Kerber KA, Jahn K, Bertholon P, Waterston J, Lee H, Bisdorff A, Strupp M (2022). Vascular vertigo and dizziness: Diagnostic criteria: consensus document of the committee for the classification of vestibular disorders of the Bárány society. J Vestib Res.

[52] Kremmyda O, Hüfner K, Flanagin VL, Hamilton DA, Linn J, Strupp M, Jahn K & Brandt T (2016) Beyond Dizziness: virtual navigation, spatial anxiety and hippocampal volume in bilateral vestibulopathy. Front Hum Neurosci. 10.3389/fnhum.2016.00139 10.3389/fnhum.2016.00139 PMC4814552 27065838

[53] Kurre A, van Gool CJAW, Bastiaenen CHG, Gloor-Juzi T, Straumann D, de Bruin ED (2009). Translation, cross-cultural adaptation and reliability of the German version of the dizziness handicap inventory. Otol Neurotol.

[54] Lacour M, Tighilet B (2010). Plastic events in the vestibular nuclei during vestibular compensation: the brain orchestration of a “deafferentation” code. Restor Neurol Neurosci.

[55] Lacroix E, Deggouj N, Salvaggio S, Wiener V, Debue M, Edwards MG (2016). The development of a new questionnaire for cognitive complaints in vertigo: the Neuropsychological Vertigo Inventory (NVI). Eur Arch Otorhinolaryngol.

[56] Lacroix E, Edwards MG, Volder AD, Noel M-P, Rombaux P, Deggouj N (2020). Neuropsychological profiles of children with vestibular loss. J Vestib Res.

[57] Lee S-U, Kim H-J, Kim J-S (2020). Bilateral vestibular dysfunction. Semin Neurol.

[58] Li Y, Chen X, Zhang Q, Xu W, Li J, Ji F, Dong Q, Chen C, Li J (2023). Effects of working memory span training on top-down attentional asymmetry at both neural and behavioral levels. Cereb Cortex.

[59] Linares R, Pelegrina S (2023). The relationship between working memory updating components and reading comprehension. Cogn Process.

[60] Löfkvist U, Almkvist O, Lyxell B, Tallberg I-M (2012). Word fluency performance and strategies in children with cochlear implants: Age-dependent effects?. Scand J Psychol.

[61] Lopez C, Blanke O (2011). The thalamocortical vestibular system in animals and humans. Brain Res Rev.

[62] Lopez C, Vibert D, Mast FW (2011). Can imagined whole-body rotations improve vestibular compensation?. Med Hypotheses.

[63] Loughrey DG, Kelly ME, Kelley GA, Brennan S, Lawlor BA (2018) Association of age-related hearing loss with cognitive function, cognitive impairment, and dementia: a systematic review and meta-analysis. JAMA Otolaryngol–Head & Neck Surg 144(2):115. 10.1001/jamaoto.2017.2513 10.1001/jamaoto.2017.2513 PMC5824986 29222544

[64] Lucieer FMP, Van Hecke R, van Stiphout L, Duijn S, Perez-Fornos A, Guinand N, Van Rompaey V, Kingma H, Joore M, van de Berg R (2020). Bilateral vestibulopathy: Beyond imbalance and oscillopsia. J Neurol.

[65] Mast FW, Preuss N, Hartmann M, Grabherr L (2014). Spatial cognition, body representation and affective processes: the role of vestibular information beyond ocular reflexes and control of posture. Front Integr Neurosci.

[66] Matotek K, Saling MM, Gates P, Sedal L (2001). Subjective complaints, verbal fluency, and working memory in mild multiple sclerosis. Appl Neuropsychol.

[67] Miller KM, Price CC, Okun MS, Montijo H, Bowers D (2009). Is the N-back task a valid neuropsychological measure for assessing working memory?. Arch Clin Neuropsychol.

[68] Mitsutake T, Sakamoto M, Horikawa E (2021). Comparing activated brain regions between noisy and conventional galvanic vestibular stimulation using functional magnetic resonance imaging. NeuroReport.

[69] Miyake A, Friedman NP, Emerson MJ, Witzki AH, Howerter A, Wager TD (2000). The unity and diversity of executive functions and their contributions to complex “Frontal Lobe” tasks: a latent variable analysis. Cogn Psychol.

[70] Moser I, Vibert D, Caversaccio MD, Mast FW (2016). Acute peripheral vestibular deficit increases redundancy in random number generation. Exp Brain Res.

[71] Moser I, Vibert D, Caversaccio MD, Mast FW (2017). Impaired math achievement in patients with acute vestibular neuritis. Neuropsychologia.

[72] Nasreddine ZS, Phillips NA, Bédirian V, Charbonneau S, Whitehead V, Collin I, Cummings JL, Chertkow H (2005). The montreal cognitive assessment, MoCA: a brief screening tool for mild cognitive impairment. J Am Geriatr Soc.

[73] Nelson JK, Reuter-Lorenz PA, Persson J, Sylvester C-YC, Jonides J (2009). Mapping interference resolution across task domains: a shared control process in left inferior frontal gyrus. Brain Res.

[74] Obermann M, Gebauer A, Arweiler-Harbeck D, Lang S, Seilheimer B, Kleinschnitz C, Diener H, Holle D, Naegel S (2023). Cognitive deficits in patients with peripheral vestibular dysfunction. Eur J Neurol.

[75] Popp P, Wulff M, Finke K, Rühl M, Brandt T, Dieterich M (2017). Cognitive deficits in patients with a chronic vestibular failure. J Neurol.

[76] R Core Team (2021) R: A language and environment for statistical computing. R Foundation for Statistical Computing, Vienna, Austria. https://www.R-project.org/

[77] Redfern MS, Talkowski ME, Jennings JR, Furman JM (2004). Cognitive influences in postural control of patients with unilateral vestibular loss. Gait Posture.

[78] Richmond LL, Morrison AB, Chein JM, Olson IR (2011). Working memory training and transfer in older adults. Psychol Aging.

[79] Rizk H, Agrawal Y, Barthel S, Bennett ML, Doherty JK, Gerend P, Gold DR, Morrill D, Oas JG, Roberts JK, Woodson E, Zapala DA, Bennett A, Shenoy AM (2018). Quality improvement in neurology: neuro-otology quality measurement set. Neurology.

[80] Robinson G, Shallice T, Bozzali M, Cipolotti L (2012). The differing roles of the frontal cortex in fluency tests. Brain.

[81] Schöne CG, Mast FW (2023). High-current galvanic vestibular stimulation impairs working memory span, but not other executive functions. Neuropsychologia.

[82] Schöne CG, Rebsamen M, Wyssen G, Rummel C, Wagner F, Vibert D, Mast FW (2022). Hippocampal volume in patients with bilateral and unilateral peripheral vestibular dysfunction. Neuroimage.

[83] Semenov YR, Bigelow RT, Xue Q-L, du Lac S, Agrawal Y (2016). Association between vestibular and cognitive function in U.S. adults: data from the national health and nutrition examination survey. J Gerontol Ser A.

[84] Shaw DJ (1967). Estimating WAIS IQ from progressive matrices scores. J Clin Psychol.

[85] Simone AN, Marks DJ, Bédard A-C, Halperin JM (2018). Low Working memory rather than ADHD symptoms predicts poor academic achievement in school-aged children. J Abnorm Child Psychol.

[86] Smith PF (2022). Hearing loss versus vestibular loss as contributors to cognitive dysfunction. J Neurol.

[87] Smith PF, Zheng Y (2013). From ear to uncertainty: Vestibular contributions to cognitive function. Front Integr Neurosci.

[88] Smith PF, Zheng Y, Horii A, Darlington CL (2005). Does vestibular damage cause cognitive dysfunction in humans?. J Vestib Res.

[89] Smith-Ray RL, Hughes SL, Prohaska TR, Little DM, Jurivich DA, Hedeker D (2013). Impact of cognitive training on balance and gait in older adults. J Gerontol B Psychol Sci Soc Sci.

[90] Smith-Ray RL, Makowski-Woidan B, Hughes SL (2014). A Randomized trial to measure the impact of a community-based cognitive training intervention on balance and gait in cognitively intact black older adults. Health Educ Behav.

[91] Smith-Ray RL, Irmiter C, Boulter K (2016). Cognitive training among cognitively impaired older adults: a feasibility study assessing the potential improvement in balance. Front Public Health.

[92] Strupp M, Kim J-S, Murofushi T, Straumann D, Jen JC, Rosengren SM, Della Santina CC, Kingma H (2017). Bilateral vestibulopathy: diagnostic criteria consensus document of the classification committee of the Bárány society1. J Vestib Res.

[93] Strupp M, Dlugaiczyk J, Ertl-Wagner BB, Rujescu D, Westhofen M, Dieterich M (2020). Vestibular disorders. Dtsch Arztebl Int.

[94] Strupp M, Bisdorff A, Furman J, Hornibrook J, Jahn K, Maire R, Newman-Toker D, Magnusson M (2022). Acute unilateral vestibulopathy/vestibular neuritis: diagnostic criteria: consensus document of the committee for the classification of vestibular disorders of the Bárány Society. J Vestib Res.

[95] Tangen GG, Engedal K, Bergland A, Moger TA, Mengshoel AM (2014). Relationships between balance and cognition in patients with subjective cognitive impairment, mild cognitive impairment, and Alzheimer disease. Phys Ther.

[96] Thürling M, Hautzel H, Küper M, Stefanescu MR, Maderwald S, Ladd ME, Timmann D (2012). Involvement of the cerebellar cortex and nuclei in verbal and visuospatial working memory: a 7T fMRI study. Neuroimage.

[97] van der Wardt V, Logan P, Hood V, Booth V, Masud T, Harwood R (2015). The association of specific executive functions and falls risk in people with mild cognitive impairment and early-stage dementia. Dement Geriatr Cogn Disord.

[98] Van Hecke R, Danneels M, Deconinck FJA, Dhooge I, Leyssens L, Van Acker E, Van Waelvelde H, Wiersema JR, Maes L (2023). A cross-sectional study on the neurocognitive outcomes in vestibular impaired school-aged children: Are they at higher risk for cognitive deficits?. J Neurol.

[99] Vaughan L, Giovanello K (2010). Executive function in daily life: Age-related influences of executive processes on instrumental activities of daily living. Psychol Aging.

[100] Vernucci S, Canet-Juric L, Richard’s MM (2023). Effects of working memory training on cognitive and academic abilities in typically developing school-age children. Psychol Res.

[101] Wang Y, Huang X, Feng Y, Luo Q, He Y, Guo Q, Feng Y, Wang H, Yin S (2022). Resting-state electroencephalography and p300 evidence: age-related vestibular loss as a risk factor contributes to cognitive decline. J Alzheimer’s Dis.

[102] Wechsler D (2012). Wechsler adult intelligence scale—fourth edition. Am Psychol Assoc.

[103] Westwood SJ, Romani C (2018). Null effects on working memory and verbal fluency tasks when applying anodal tDCS to the inferior frontal gyrus of healthy participants. Front Neurosci.

[104] Yardley L (2001). Interference between postural control and mental task performance in patients with vestibular disorder and healthy controls. J Neurol Neurosurg Psychiatry.

[105] Yogev-Seligmann G, Hausdorff JM, Giladi N (2008). The role of executive function and attention in gait. Mov Disord.

[106] Zhang Q, Li Y, Zhao W, Chen X, Li X, Du B, Deng X, Ji F, Wang C, Xiang Y-T, Dong Q, Jaeggi SM, Chen C, Song Y, Li J (2020). ERP evidence for the effect of working memory span training on working memory maintenance: A randomized controlled trial. Neurobiol Learn Mem.

[107] Zimmermann P, Fimm B (2002). Testbatterie zur Aufmerksamkeitsprüfung (TAP).

