# Supplementary material for: Executive functions in patients with bilateral and unilateral peripheral vestibular dysfunction
Source: J Neurol. 2024 Mar 11;271(6):3291–308. doi: 10.1007/s00415-024-12267-7 (PMC11136862; doi:10.1007/s00415-024-12267-7)
Supplement: Supplementary file 1 — Supplementary file1 (Pdf 424 KB) [file 415_2024_12267_MOESM1_ESM.pdf]

Supplementary Material

Article title:

Executive functions in patients with bilateral and unilateral peripheral vestibular dysfunction

Journal name:

Journal of Neurology

Author names:

Schöne Corina G. [a, b *]*, Vibert Dominique* [b]*, Mast Fred W.* [a]

Author affiliations:

[a] Department of Psychology, University of Bern, Bern, Switzerland.

[b] Department of Otorhinolaryngology, Head and Neck Surgery, Inselspital, University Hospital Bern, University of Bern, Bern, Switzerland.

Corresponding author:

** Corresponding author. Department of Psychology, University of Bern, Fabrikstrasse 8, 3012 Bern, Switzerland. E-mail address: corina.schoene@unibe.ch*

Supplementary Table S1

Pairwise matches of patients with peripheral vestibular dysfunction and their respective healthy controls

Patients with bilateral PVD				Matched bilateral healthy controls			
	Age	Sex	Education[a]		Age	Sex	Education[a]
Bilateral patient 1	25	f	2	Bilateral control 1	28	f	2
Bilateral patient 2	33	f	3	Bilateral control 2	31	f	6
Bilateral patient 3	34	f	0	Bilateral control 3	38	f	2
Bilateral patient 4	34	f	2	Bilateral control 4	38	f	2
Bilateral patient 5	38	f	2	Bilateral control 5	42	f	4
Bilateral patient 6	52	f	2	Bilateral control 6	51	f	2
Bilateral patient 7	53	f	2	Bilateral control 7	52	f	2
Bilateral patient 8	54	f	3	Bilateral control 8	59	f	3
Bilateral patient 9	58	f	2	Bilateral control 9	58	f	2
Bilateral patient 10	61	f	1	Bilateral control 10	61	f	2
Bilateral patient 11	61	f	2	Bilateral control 11	63	f	3
Bilateral patient 12	64	f	2	Bilateral control 12	63	f	2
Bilateral patient 13	72	f	3	Bilateral control 13	70	f	2
Bilateral patient 14	73	f	2	Bilateral control 14	73	f	1
Bilateral patient 15	18	m	1	Bilateral control 15	20	m	1
Bilateral patient 16	22	m	4	Bilateral control 16	20	m	4
Bilateral patient 17	25	m	3	Bilateral control 17	28	m	3
Bilateral patient 18	33	m	6	Bilateral control 18	31	m	5
Bilateral patient 19	37	m	2	Bilateral control 19	37	m	4
Bilateral patient 20	45	m	7	Bilateral control 20	48	m	6
Bilateral patient 21	45	m	2	Bilateral control 21	40	m	2
Bilateral patient 22	49	m	2	Bilateral control 22	48	m	2
Bilateral patient 23	49	m	3	Bilateral control 23	46	m	2
Bilateral patient 24	50	m	1	Bilateral control 24	46	m	2
Bilateral patient 25	50	m	1	Bilateral control 25	54	m	3
Bilateral patient 26	55	m	4	Bilateral control 26	54	m	5
Bilateral patient 27	58	m	3	Bilateral control 27	58	m	2
Bilateral patient 28	60	m	7	Bilateral control 28	61	m	7
Bilateral patient 29	63	m	2	Bilateral control 29	66	m	2

	Age	Sex	Education[a]		Age	Sex	Education[a]
Bilateral patient 30	64	m	2	Bilateral control 30	66	m	2
Bilateral patient 31	68	m	6	Bilateral control 31	69	m	6
Bilateral patient 32	73	m	2	Bilateral control 32	74	m	2
Bilateral patient 33	77	m	2	Bilateral control 33	76	m	2
Bilateral patient 34	77	m	4	Bilateral control 34	75	m	3

Patients with chronic unilateral PVD				Matched chronic unilateral healthy controls			
	Age	Sex	Education[a]		Age	Sex	Education[a]
Chronic unilateral patient 1	42	f	2	Chronic unilateral control 1	38	f	2
Chronic unilateral patient 2	46	f	2	Chronic unilateral control 2	42	f	4
Chronic unilateral patient 3	53	f	2	Chronic unilateral control 3	51	f	2
Chronic unilateral patient 4	55	f	2	Chronic unilateral control 4	52	f	2
Chronic unilateral patient 5	56	f	2	Chronic unilateral control 5	58	f	2
Chronic unilateral patient 6	56	f	2	Chronic unilateral control 6	59	f	3
Chronic unilateral patient 7	59	f	2	Chronic unilateral control 7	61	f	2
Chronic unilateral patient 8	59	f	3	Chronic unilateral control 8	63	f	3
Chronic unilateral patient 9	60	f	2	Chronic unilateral control 9	63	f	2
Chronic unilateral patient 10	62	f	2	Chronic unilateral control 10	66	f	3
Chronic unilateral patient 11	68	f	1	Chronic unilateral control 11	70	f	2
Chronic unilateral patient 12	77	f	1	Chronic unilateral control 12	73	f	1
Chronic unilateral patient 13	31	m	3	Chronic unilateral control 13	28	m	3
Chronic unilateral patient 14	44	m	6	Chronic unilateral control 14	40	m	2
Chronic unilateral patient 15	44	m	6	Chronic unilateral control 15	46	m	2
Chronic unilateral patient 16	47	m	6	Chronic unilateral control 16	48	m	6
Chronic unilateral patient 17	48	m	3	Chronic unilateral control 17	48	m	2
Chronic unilateral patient 18	49	m	5	Chronic unilateral control 18	51	m	6

	Age	Sex	Education[a]		Age	Sex	Education[a]
Chronic unilateral patient 19	51	m	5	Chronic unilateral control 19	54	m	5
Chronic unilateral patient 20	57	m	2	Chronic unilateral control 20	58	m	2
Chronic unilateral patient 21	57	m	2	Chronic unilateral control 21	54	m	3
Chronic unilateral patient 22	58	m	6	Chronic unilateral control 22	55	m	6
Chronic unilateral patient 23	64	m	1	Chronic unilateral control 23	66	m	2
Chronic unilateral patient 24	66	m	3	Chronic unilateral control 24	66	m	2
Chronic unilateral patient 25	68	m	3	Chronic unilateral control 25	67	m	3
Chronic unilateral patient 26	70	m	2	Chronic unilateral control 26	69	m	6
Chronic unilateral patient 27	72	m	2	Chronic unilateral control 27	75	m	3
Chronic unilateral patient 28	72	m	3	Chronic unilateral control 28	74	m	2
Chronic unilateral patient 29	75	m	3	Chronic unilateral control 29	76	m	2

Patients with acute unilateral PVD				Matched acute unilateral healthy controls			
	Age	Sex	Education[a]		Age	Sex	Education[a]
Acute unilateral patient 1	24	f	5	Acute unilateral control 1	25	f	4
Acute unilateral patient 2	27	f	2	Acute unilateral control 2	28	f	2
Acute unilateral patient 3	32	f	7	Acute unilateral control 3	32	f	7
Acute unilateral patient 4	41	f	4	Acute unilateral control 4	42	f	4
Acute unilateral patient 5	57	f	3	Acute unilateral control 5	58	f	2
Acute unilateral patient 6	59	f	2	Acute unilateral control 6	61	f	2
Acute unilateral patient 7	24	m	4	Acute unilateral control 7	20	m	4
Acute unilateral patient 8	33	m	5	Acute unilateral control 8	31	m	5
Acute unilateral patient 9	37	m	5	Acute unilateral control 9	37	m	4
Acute unilateral patient 10	41	m	2	Acute unilateral control 10	40	m	2
Acute unilateral patient 11	47	m	3	Acute unilateral control 11	46	m	2
Acute unilateral patient 12	49	m	3	Acute unilateral control 12	46	m	2
Acute unilateral patient 13	51	m	1	Acute unilateral control 13	48	m	2
Acute unilateral patient 14	56	m	2	Acute unilateral control 14	54	m	3
Acute unilateral patient 15	57	m	6	Acute unilateral control 15	55	m	6
Acute unilateral patient 16	57	m	4	Acute unilateral control 16	54	m	5
Acute unilateral patient 17	58	m	3	Acute unilateral control 17	58	m	2
Acute unilateral patient 18	61	m	3	Acute unilateral control 18	66	m	2

	Age	Sex	Education[a]		Age	Sex	Education[a]
Acute unilateral patient 19	73	m	3	Acute unilateral control 19	74	m	2
Acute unilateral patient 20	74	m	5	Acute unilateral control 20	75	m	3

Note. [a] Education measured as maximal education reached. maximal value = 7.

Supplementary Table S2

Detailed medical information of diagnosis, semicircular function, otolithic function, and degree of vestibular deafferentation of individual patients with peripheral vestibular dysfunction

Patients with bilateral PVD						
	Diagnosis	Semicircular function		Otolithic function		Degree of vestibular deafferentation[a]
		Calorics	v-HIT lateral/vertical SCC	c- VEMP	o-VEMP	
1	Vestibular neuritis R	HR	Pathological	Absent	Absent	Complete
	Vestibular schwannoma L	AL	Pathological	Absent	Absent	
2	Vestibular schwannoma R	AR	Normal	Absent	Present	Incomplete
	Idiopathic L	HL	Normal	Present	Present	
3	Meningitis	AR	Pathological	Absent	Absent	Complete
		AL	Pathological	Absent	Absent	
4	Idiopathic R	AR	Pathological	Present	Absent	Incomplete
	Idiopathic L	AL	Pathological	Present	Absent	
5	Idiopathic R	AR	ND	ND	ND *	Incomplete
	Idiopathic L	AL	ND	ND	ND *	
6	Meningitis	AR	Pathological	Absent	ND *	Complete
		AL	Pathological	Absent	ND *	
7	Gentamycin ototoxicity	AR	Pathological	Absent	Absent	Complete
		AL	Pathological	Absent	Absent	
8	Idiopathic R	AR	Pathological	NV	ND *	Incomplete
	Idiopathic L	AL	Pathological	NV	ND *	
9	Meningitis	AR	Pathological	Absent	ND *	Complete
		AL	Pathological	Absent	ND *	
10	Endolymphatic hydrops R	AR	Pathological	Absent	Absent	Incomplete
	Endolymphatic hydrops L	AL	Normal	Absent	Absent	
11	Idiopathic R	AR	Normal	Present	Present	Incomplete
	Idiopathic L	HL	Normal	Absent	Present	
12	Meningitis	AR	Pathological	Absent	ND *	Complete
		AL	Pathological	Absent	ND *	
13	Meningitis	AR	Pathological	Absent	ND *	Complete
		AL	Pathological	Absent	ND *	
14	Meningitis	AR	Pathological	Absent	ND *	Complete
		AL	Pathological	Absent	ND *	
15	Idiopathic R	AR	Pathological	Absent	ND *	Incomplete
	Idiopathic L	AL	Pathological	Absent	ND *	
16	Idiopathic R	AR	Pathological	Absent	Absent	Incomplete
	Idiopathic L	AL	Pathological	Absent	Absent	

17	Idiopathic R	AR	Pathological	ND	ND *	Incomplete
	Idiopathic L	AL	Pathological	ND	ND *	
18	Idiopathic R	HR	Normal	Present	Present	Incomplete
	Idiopathic L	AL	Pathological	Present	Absent	
19	Idiopathic R	AR	Normal	Present	Absent	Incomplete
	Idiopathic L	AL	Pathological	Present	Absent	
20	Vestibular neuritis R	AR	Pathological	Absent	Absent	Incomplete
	Vestibular neuritis L	AL	Pathological	Absent	Absent	
21	Endolymphatic hydrops R	AR	Pathological	Absent	ND *	Incomplete
	Endolymphatic hydrops L	AL	Pathological	Absent	ND *	
22	Vestibular schwannoma R	AR	Pathological	Absent	Absent	Complete
	Idiopathic L	AL	Pathological	Absent	Absent	
23	Vestibular schwannoma R	AR	Pathological	Absent	Absent	Incomplete
	Idiopathic L	HL	Normal	Present	Absent	
24	Idiopathic R	AR	Pathological	Absent	ND *	Incomplete
	Idiopathic L	AL	Pathological	Absent	ND *	
25	Idiopathic R	AR	Pathological	Absent	Absent	Complete
	Idiopathic L	AL	Pathological	Absent	Absent	
26	Idiopathic R	AR	Pathological	Absent	Present	Incomplete
	Idiopathic L	AL	Normal	Present	Absent	
27	Meningitis	AR	Pathological	Absent	ND *	Complete
		AL	Pathological	Absent	ND *	
28	Idiopathic R	AR	Pathological	Absent	Present	Incomplete
	Idiopathic L	AL	Pathological	Absent	Absent	
29	Vestibular neuritis L	HR	Normal	Absent	Present	Incomplete
	Idiopathic R	HL	Normal	Absent	Present	
30	Endolymphatic hydrops R	AR	Pathological	Absent	Absent	Incomplete
	Endolymphatic hydrops L	AL	Pathological	Present	Absent	
31	Meningitis	AR	Pathological	Absent	Absent	Complete
		AL	Pathological	Absent	Absent	
32	Idiopathic R	HR	Normal	Absent	Present	Incomplete
	Idiopathic L	HL	Normal	Absent	Present	
33	Idiopathic R	AR	Pathological	Absent	Absent	Complete
	Idiopathic L	AL	Pathological	Absent	Absent	
34	Idiopathic R	AR	Pathological	Present	Absent	Incomplete
	Idiopathic L	AL	Pathological	Absent	Absent	

Patients with chronic unilateral PVD						
	Diagnosis	Semicircular function		Otolithic function		Degree of vestibular deafferentation[a]
		Calorics	v-HIT lateral/vertical SCC	c- VEMP	o-VEMP	
1	Vestibular schwannoma L	AL	Pathological	Absent	Present	Incomplete
2	Vestibular neurectomy L	AL	Pathological	Absent	Absent	Complete
3	Vestibular schwannoma L	AL	Pathological	Absent	Absent	Complete
4	Vestibular schwannoma R	HR	Normal	Present	Absent	Incomplete
5	Vestibular schwannoma L	HL	Pathological	Absent	Present	Incomplete
6	Vestibular schwannoma R	AR	Pathological	Absent	Absent	Complete
7	Vestibular schwannoma L	HL	Normal	Absent	ND	Incomplete
8	Vestibular schwannoma R	AR	Pathological	Absent	Absent	Complete
9	Endolymphatic hydrops R	HR	Normal	Present	Present	Incomplete
10	Vestibular schwannoma R	HR	Normal	Absent	Absent	Incomplete
11	Vestibular schwannoma L	AL	Pathological	Absent	Absent	Complete
12	Vestibular schwannoma R	AR	Normal	Absent	Absent	Incomplete
13	Vestibular schwannoma L	AL	Normal	Absent	Absent	Incomplete
14	Vestibular schwannoma R	HR	Pathological	Absent	Absent	Complete
15	Vestibular neuritis R	HR	Normal	Present	Present	Incomplete
16	Zoster oticus L	AL	Pathological	Absent	Absent	Complete
17	Vestibular neurectomy R	AR	Pathological	Absent	Present	Incomplete
18	Vestibular schwannoma L	AL	Pathological	Absent	Absent	Complete
19	Vestibular schwannoma R	HR	Pathological	Absent	Absent	Complete
20	Vestibular schwannoma L	AL	Pathological	Absent	Present	Incomplete
21	Vestibular schwannoma L	HL	NV	NV	NV	Incomplete
22	Vestibular neuritis R	AR	Pathological	Absent	Present	Incomplete

23	Vestibular schwannoma L	HL	Normal	Absent	Present	Incomplete
24	Labyrinthectomy L	AL	Pathological	Absent	Absent	Complete
25	Endolymphatic hydrops R	HR	Normal	NV	Present	Incomplete
26	Vestibular schwannoma R	AR	Pathological	Absent	Absent	Complete
27	Vestibular neurectomy L	AL	Pathological	Absent	Absent	Complete
28	Endolymphatic hydrops R	HR	Normal	Absent	ND	Incomplete
29	Meningitis	HL	Normal	Present	Present	Incomplete

Patients with acute unilateral PVD						
	Diagnosis	Semicircular function		Otolithic function		Degree of vestibular deafferentation[a]
		Calorics	v-HIT lateral/vertical SCC	c- VEMP	o-VEMP	
1	Vestibular neuritis L	AL	Pathological	Absent	Absent	Complete
2	Vestibular neuritis L	HL	Pathological	Absent	Present	Incomplete
3	Vestibular neuritis R	HR	Normal	Present	Present	Incomplete
4	Vestibular neuritis L	HL	Normal	Present	Absent	Incomplete
5	Vestibular neuritis R	HR	ND	Absent	Present	Incomplete
6	Vestibular neuritis L	AL	Normal	Present	Present	Incomplete
7	Vestibular neuritis L	HL	Pathological	Absent	Absent	Complete
8	Vestibular neuritis L	HL	Normal	Present	Present	Incomplete
9	Vestibular neuritis R	HR	Normal	Present	Present	Incomplete
10	Idiopathic L	HL	Pathological	Absent	Absent	Complete
11	Vestibular neuritis R	HR	Normal	Present	Absent	Incomplete
12	Idiopathic L	HL	Pathological	Present	Present	Incomplete
13	Vestibular neuritis R	HR	Normal	Absent	Absent	Incomplete
14	Vestibular neuritis R	HR	Normal	Absent	Absent	Incomplete
15	Vestibular neuritis R	HR	Normal	Absent	Absent	Incomplete
16	Vestibular neuritis R	HR	Normal	Present	Present	Incomplete
17	Vestibular neuritis R	AR	Pathological	Present	Present	Incomplete
18	Vestibular neuritis R	HR	Pathological	Absent	Present	Incomplete
19	Vestibular neuritis R	HR	Normal	Present	Absent	Incomplete
20	Vestibular neuritis R	AR	Pathological	Present	NV	Incomplete

Note. PVD peripheral vestibular dysfunction, v-HIT video head impulse test, *SCC* semicircular canal, *c-VEMP* cervical vestibular evoked myogenic potentials, *o-VEMP* ocular vestibular evoked myogenic potentials, *L* left, *R* right, *A* areflexia, *H* hyporeflexia, *NV* not available, *ND* not done, *ND ** o-VEMP not performed within cochlear implanted patients.

[a] Vestibular deafferentation degree: complete = pathological semicircular canal & otolithic functions. incomplete = pathological semicircular canal function (caloric test), but intact otolithic functions.
